# Optimized synthesis of suvorexant and determination of eight residual solvents by headspace gas chromatography

**DOI:** 10.1039/d5ra06779k

**Published:** 2025-10-30

**Authors:** Chenshuo Jia, Zixing Yu, Yuanyuan Liu, Xu Wang, Qiao Wang, Jingjing Zhao, Weiguo Shi, Aiping Zheng

**Affiliations:** a State Key Laboratory of National Security Specially Needed Medicines Beijing 100850 China apzheng@163.com shiwg1988@126.com; b College of Pharmacy, Hebei Medical University Shijiazhuang 050000 China

## Abstract

This study presents an optimized synthetic pathway for suvorexant and establishes a robust method for the simultaneous determination of residual solvents. The synthesis commenced with chiral precursors, specifically (*R*)-3-(BOC-amino)butyric acid and *N*-benzyl glycine ethyl ester, employing a fragment splicing strategy. The target compound was synthesized through a sequence of nucleophilic reactions, BOC deprotection, cyclization, reduction, BOC protection, affinity substitution, and subsequent nucleophilic reactions, thereby circumventing the need for chiral separation. The post-treatment process was refined *via* recrystallization to yield the active pharmaceutical ingredient (API). For residual solvent analysis, a headspace gas chromatography (HS-GC) method was developed, utilizing a DB-624 capillary column (30 m × 0.53 mm, 3 μm) with programmed temperature control. The chromatographic conditions included an inlet temperature of 220 °C and a detector temperature of 280 °C, with detection *via* hydrogen flame ionization. The final product exhibited a purity of 99.92% and an overall yield of 65%. The HS-GC method demonstrated excellent resolution (*R* > 1.5) for eight residual solvents, including *n*-heptane, with linearity (*r* > 0.990) across the specified range, average spiked recoveries between 85–115%, and relative standard deviations (RSD) below 5.0%. The optimized synthesis is characterized by cost-effectiveness, operational simplicity, and high yield, rendering it suitable for industrial-scale production. The established HS-GC method exhibits high specificity and sensitivity, making it a reliable approach for residual solvent quantification.

## Abstract 2

This study presents an optimized synthetic pathway for suvorexant and establishes a robust method for the simultaneous determination of residual solvents.

## Graphical abstract

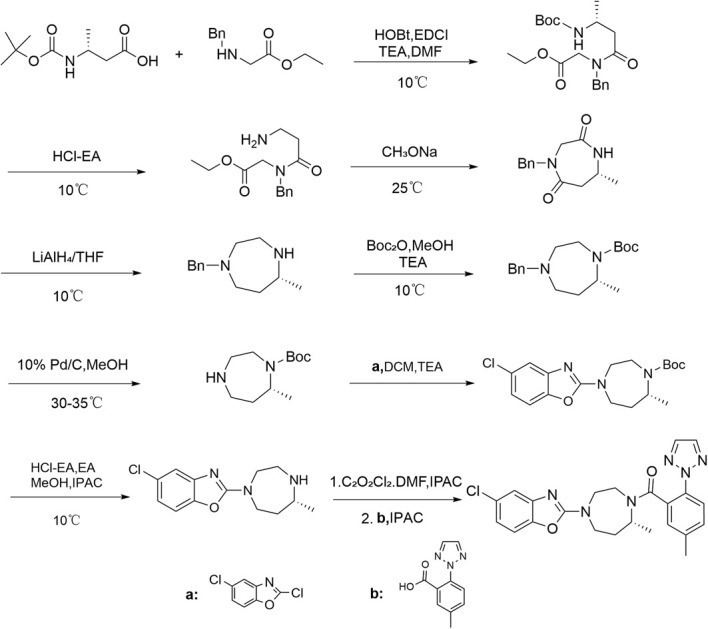

## 1. Introduction

Insomnia, a prevalent sleep disorder, is clinically defined by persistent difficulties in sleep initiation, maintenance, or impaired sleep quality, accompanied by significant daytime impairments including fatigue, cognitive dysfunction, impaired concentration, irritability, anxiety, and depressive symptoms.^1–3^ Epidemiological studies estimate that this condition affects approximately 30% of the global population, manifesting either as a primary disorder or comorbidity with psychiatric, metabolic, and cardiovascular conditions.^4^ The substantial socioeconomic impact of insomnia, with annual healthcare costs reaching billions of dollars, underscores the critical need for effective therapeutic interventions. Orexins (hypocretins), a class of neuropeptides synthesized in the lateral hypothalamus, play a pivotal role in the regulation of sleep–wake cycles and maintenance of wakefulness. Two distinct isoforms have been characterized: orexin-A (OX-A), a 33-amino acid peptide, and orexin-B (OX-B), comprising 28 amino acids.^5,6^ Comparative biochemical analyses reveal that OX-A exhibits greater biological stability, higher tissue and plasma concentrations, and enhanced lipophilicity compared to OX-B, facilitating more efficient blood-brain barrier penetration. These pharmacological properties have established OX-A as a primary focus in clinical research. Notably, experimental evidence from rodent, canine, and human studies has demonstrated a direct correlation between reduced orexin levels and the pathogenesis of narcolepsy.^7–9^ These findings suggest that pharmacological modulation of orexin receptor activity through specific antagonists may represent a promising therapeutic strategy for sleep disorders, particularly insomnia.

**Fig. 1:**
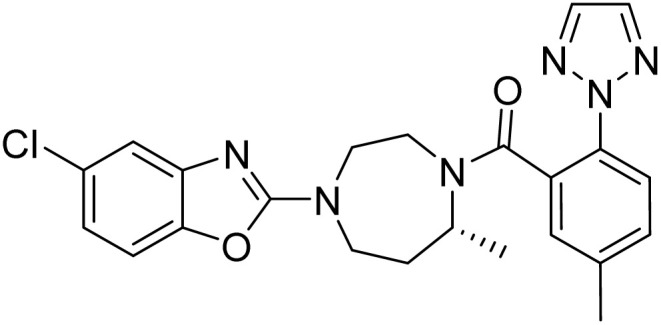
Structure of suvorexant.

**Fig. 2:**
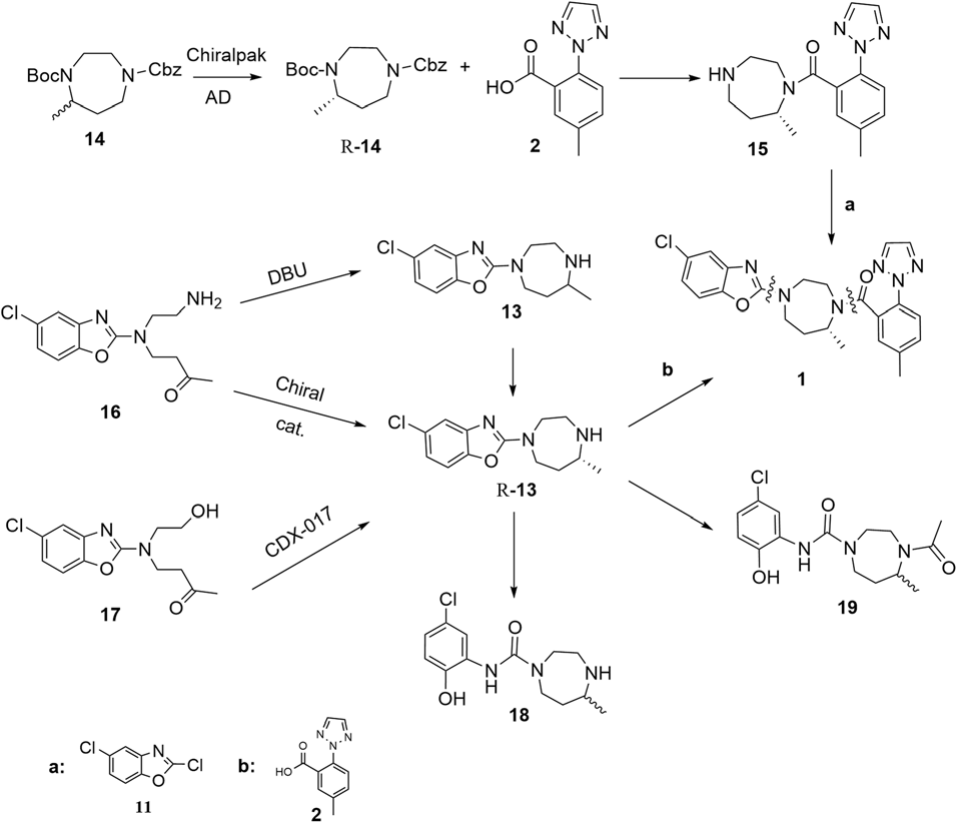
Summarizes the synthetic route of suvorexant based on literature reports.

Suvorexant (Fig. 1), the first clinically approved orexin receptor antagonist, demonstrates several advantageous pharmacological characteristics, including rapid absorption, well-defined metabolic pathways, a high safety margin, robust therapeutic efficacy, and a low potential for dependence. Furthermore, recent studies indicate that suvorexant may play a role in modulating addictive behaviors, thereby broadening its potential therapeutic applications beyond the treatment of insomnia.^10–13^ Consequently, suvorexant not only serves as a pivotal compound for mechanistic investigations but also exhibits significant clinical promise and substantial commercial development potential. In recent years, several protocols have been developed for the synthesis of suvorexant Fig. 2. The route developed by Cox *et al.* in 2010.^14^ Central to this approach was the synthesis of the core diazepane *R*-14, which was afforded by a preparative chiral high-performance liquid chromatography (HPLC) separation of orthogonally protected racemic 14 removal of the Boc protecting group, coupling with compound 2, and hydrogenolysis of the Cbz group yielded compound 15. Finally, in the presence of potassium carbonate, a condensation reaction with 2,5-dichloro-1,3-benzoxazole 11 is carried out to obtain compound 1. This route utilizes column chromatography and chiral high-performance liquid chromatography separation methods, which are not advantageous for large-scale preparation. The large-scale synthesis of suvorexant was reported by baxter.^15^ The key intermediate *R*-13 was obtained through a classical resolution method, whereas the racemate 13 was synthesized *via* reductive amination of compound 16, followed by condensation with compound 2 to yield compound 1. However, this synthetic approach not only produced the desired racemate 13 but also led to the formation of impurities 18 and 19, which proved resistant to subsequent purification efforts, The existing methodologies present notable challenges in quality control. Strotman *et al.*^16^ pioneered the asymmetric reductive amination of dialkyl ketones with alkyl amines, employing a novel chiral ruthenium-based transfer hydrogenation catalyst. This approach yielded the bicyclic nitrogen-containing compound *R*-13 with a 97% yield and high enantiomeric purity (94.5% enantiomeric excess). However, the high cost of the ruthenium catalyst and the insufficient optical purity of the product for pharmaceutical applications necessitate additional chiral resolution steps. Concurrently, Mangion *et al.*^17^ developed an alternative strategy for suvorexant synthesis utilizing the biocatalyst CDX-017.This transamination reaction of compound 17 achieved a high conversion rate and exceptional enantiomeric purity (>99% enantiomeric excess). Despite the efficient construction of the chiral seven-membered bicyclic nitrogen ring, the reaction generated substantial byproducts from intramolecular amination cyclization, resulting in a suboptimal yield. Furthermore, the non-commercial availability of CDX-017 and the sensitivity of the strong base to moisture and air impose significant practical limitations (Fig. 2).

This study developed an optimized process route amenable to industrial scale-up, effectively circumventing the requirement for chiral column separation. The methodology employs a one-pot synthesis approach for the key intermediate, thereby obviating the necessity for intermediate purification and impurity removal steps. The final product was obtained in high-purity crystalline form through a recrystallization process, demonstrating the efficiency and practicality of the proposed synthetic route. All the above synthetic routes for suvorexant involve the use of various organic solvents, including isopropyl acetate (IPAC), *n*-heptane (*n*-Hept), *N*,*N*-dimethylformamide (DMF), triethylamine (TEA), ethyl acetate (EA), dichloromethane (DCM), methanol (MeOH), tetrahydrofuran (THF), acetonitrile (CAN), chloroform (CHCl_3_), dimethyl sulfoxide (DMSO), *etc.* Therefore, how to detect the content of the above-mentioned solvents in the final product has become an important part of controlling product quality.

The optimization of chemical and formulation processes is an integral component of drug development and manufacturing. The selection of organic solvents is a critical determinant, significantly impacting key parameters such as reaction yield, impurity profile, solid-state characteristics, product stability, and the overall quality of the pharmaceutical product.^18,19^ As a result, residual solvent control has become a critical step in ensuring the safety and compliance of pharmaceutical products. Static headspace-gas chromatography (HS-GC) technology, due to its efficiency and reliability, has become the method specified by the United States Pharmacopeia (USP) Chapter 〈467〉 for residual solvent testing and is widely used in drug development and production.^20–22^

Currently, no published literature systematically evaluates the levels and control strategies of residual solvents in the synthesis of suvorexant. Considering that various organic solvents, it is especially critical to strictly monitor the residual amounts of DCM, DMF, and THF due to their potential toxicity or carcinogenicity,^23,24^ developing a multi-solvent simultaneous detection method that aligns with process improvements is urgently needed. Existing studies indicate that even after vacuum drying, trace amounts of certain solvents may remain in the final product, posing potential risks to the drug's therapeutic efficacy and stability.^25^ The present investigation utilized static headspace-gas chromatography (HS-GC) methodology to develop and validate a robust analytical protocol for residual solvent quantification, thereby offering essential technical support for both quality assurance in product manufacturing and optimization of process development.

## 2. Results and discussion

### 2.1 Synthesis of suvorexant

The synthesis of suvorexant was accomplished through a fragment coupling strategy, which demonstrated a cumulative yield of 65% over nine sequential steps, with each step exhibiting high efficiency. The procedure is characterized by its operational simplicity, reproducibility, and robustness. To minimize the generation of chiral impurities, chiral starting materials were strategically employed. Specifically, (*R*)-3-((*tert*-butoxycarbonyl)amino)butanoic acid and ethyl (*R*)-*N*-benzyl-*N*-(3-((*tert*-butoxycarbonyl)amino)butanoyl) glycinate were selected as the foundational precursors for the synthesis.

Suvorexant, the first clinically approved dual orexin receptor antagonist, necessitates the development of efficient synthetic methodologies to support both clinical applications and industrial-scale manufacturing. This study presents an optimized, scalable synthetic route derived from an established fragment coupling strategy. Key process improvements were achieved through reaction condition optimization and enhanced post-reaction processing protocols. Specifically, the BOC deprotection of compound 4 was optimized by employing a 4.0 mol per L hydrochloric acid–ethyl acetate solution at a controlled temperature of 50 ± 2 °C, resulting in a significant reduction of reaction time from 6 hours to 2.5 hours. Subsequent isolation of compound 5 as its hydrochloride salt was accomplished through aqueous extraction, followed by organic solvent washing to eliminate non-polar impurities. The final product was obtained through pH adjustment, extraction, and concentration, yielding enhanced purity while mitigating equipment corrosion risks associated with acidic residues.^26^ In the synthesis of compound 7, a 40% reduction in reaction time was achieved compared to previously reported methods. The de-BOC reaction of compound 11 was conducted using 4.0 mol per L hydrochloric acid–ethyl acetate solution, with subsequent purification through recrystallization from methanol–isopropyl acetate, affording a high-purity intermediate (>99%) with minimal impurity content (<0.2%).

In the final crystallization step, suvorexant was synthesized through a gradient cooling protocol, comprising dissolution at 80 °C, seeded crystallization at 65 °C, and gradual crystallization at 45 °C, followed by *n*-heptane washing. This optimized procedure yielded a product of exceptional purity (99.92% by HPLC) with an overall yield of 50%, representing a 20% enhancement compared to previously reported methods.

**Scheme 1:**
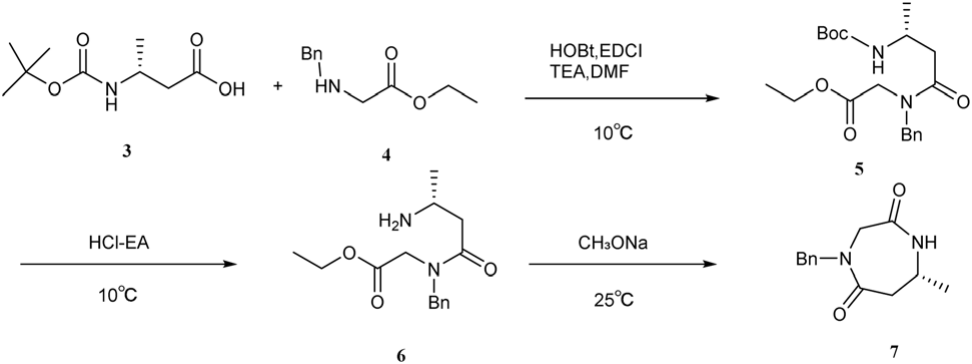
Construction of seven-membered rings.

**Scheme 2:**
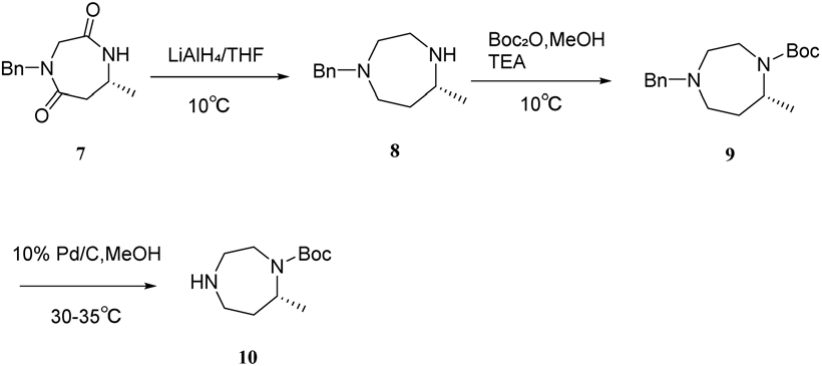
The process of introduction and deprotection of protecting groups.

**Scheme 3:**
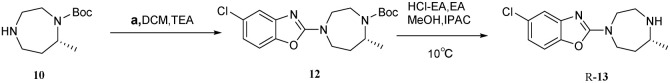
Formation of the seven-membered diazacyclic ring system.

**Scheme 4:**
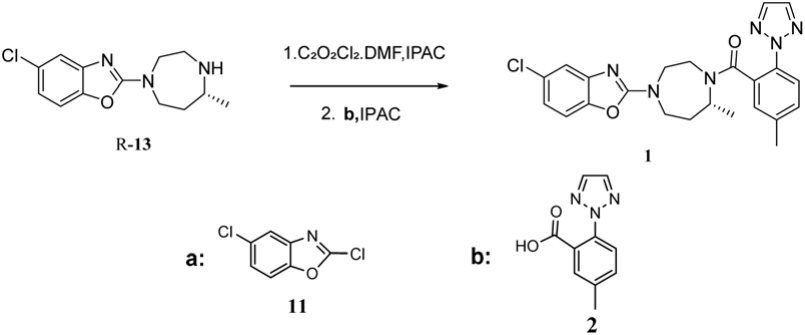
Synthesis of suvorexant.

The synthetic route demonstrates significant advancements, including the utilization of recyclable solvents (ethyl acetate and *n*-heptane) Schemes 1–4*in lieu* of highly toxic reagents such as methyl vinyl ketone, thereby aligning with green chemistry principles. Additionally, the implementation of continuous rotary evaporation and gradient crystallization reduced processing time by 30% and improved atom economy to 65%, enhancing overall process efficiency. The mild reaction conditions (≤80 °C), coupled with a simplified post-treatment protocol that eliminates the need for specialized equipment, render this method highly amenable to scale-up in reactors exceeding 50 L, thereby establishing a robust framework for the industrial production of suvorexant API.

### 2.2 Establishment of residual solvent determination method and sample analysis

#### 2.2.1 System suitability test

**Fig. 3:**
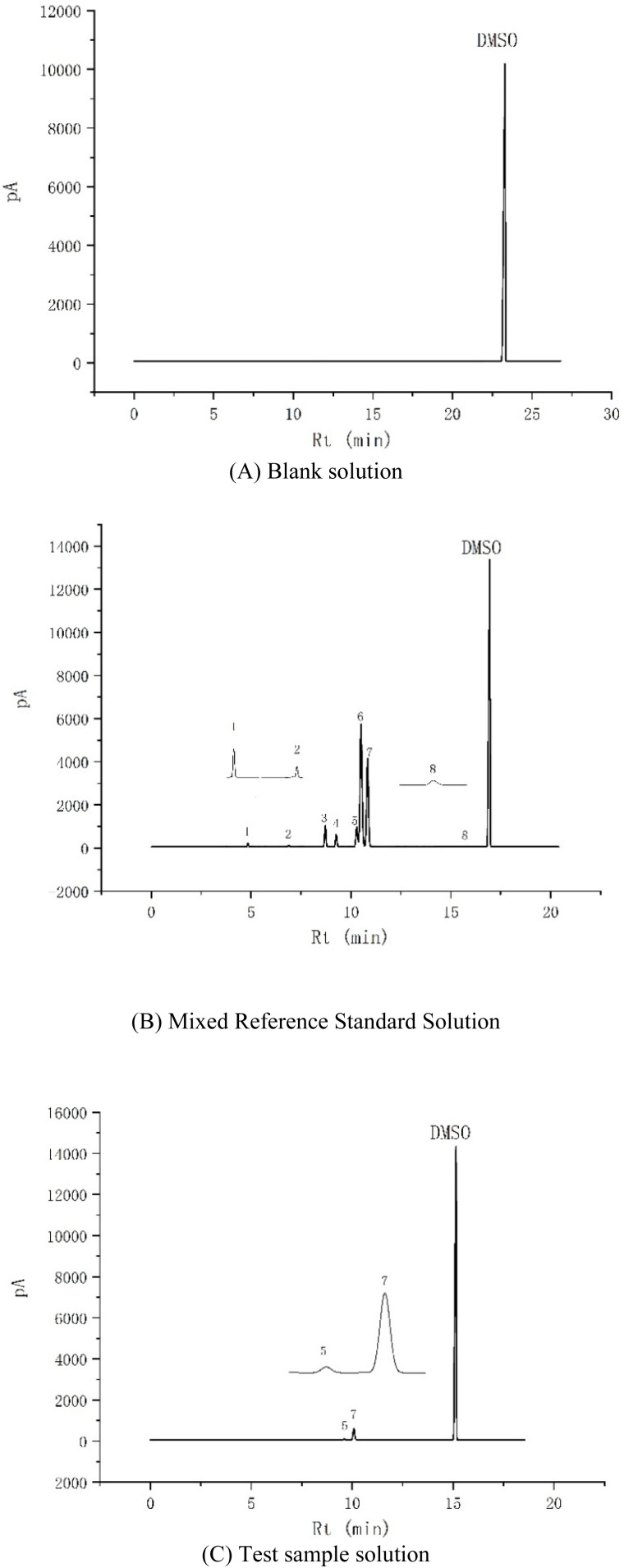
Gas chromatogram of blank solvent (A), standard solution (B) and test solution (C). (1) Methanol (2) dichloromethane (3) ethyl acetate (4) tetrahydrofuran (5) isopropyl acetate (6) triethylamine (7) *n*-heptane (8) *N*,*N*-dimethylformamide.

Five aliquots of a mixed reference solution were subjected to headspace sampling, and chromatograms were recorded. The reproducibility, resolution, theoretical plates, and tailing factor were evaluated. The results indicated that the peaks for all eight solvents were well-formed (Fig. 3), with the RSD of peak areas for each solvent not exceeding 5%. The resolution between adjacent peaks was greater than 1.5, and the theoretical plates for each component were no less than 5000.

#### 2.2.2 Method validation

##### 2.2.2.1 Specificity evaluation

Headspace sampling was performed with blank solvent (dimethyl sulfoxide, DMSO), mixed reference solution, and sample solution, and the chromatograms were recorded (Fig. 3B). As shown in the figure, the blank solvent did not interfere with the analysis of the eight organic solvents.

##### 2.2.2.2 Limit of detection and limit of quantification test

The reference solution was serially diluted to low concentrations, and the mass concentration corresponding to signal-to-noise ratios (S/N) of 3 and 10 were determined as the limit of detection and limit of quantification, respectively. The results are shown in Table 1.

**Table 1:** Linear range, the limit of quantitation and determination of 8 kinds of residual solvent

Solvent	Regression equation	*r*	*ρ*_linear range_/(mg L^−1^)	*ρ*_LOD_/(μg L^−1^)	*ρ*_LOQ_/(μg L^−1^)
MeOH	*A* = 1.2936*ρ* − 5.8610	0.9997	12.090–1208.8	3.627	12.090
DCM	*A* = 1.6775*ρ* − 0.9463	0.9995	0.981–246.320	2.153	7.177
EA	*A* = 5.7979*ρ* − 27.6728	0.9995	3.030–2002.720	0.909	3.030
THF	*A* = 10.6053*ρ* − 9.0978	0.9995	2.885–304.160	0.866	2.885
IPAC	*A* = 6.3233*ρ* − 9.8571	0.9986	3.012–2010.920	9.04	3.012
TEA	*A* = 36.8768*ρ* − 56.9641	0.9990	0.599–2006.480	0.180	0.599
*n*-Heptane	*A* = 72.2685*ρ* + 698.0213	0.9988	0.599–2017.880	0.091	0.302
DMF	*A* = 0.1892*ρ* − 0.3126	0.9989	34.548–355.440	10.364	34.548

##### 2.2.2.3 Linearity evaluation

A series of reference solutions was prepared by diluting the reference solution to 200%, 160%, 120%, 100%, 80%, 40%, and the limit of quantification (Table 1) for each solvent. An aliquot of 5.0 mL from each solution was precisely measured and placed in a 20 mL headspace vial, sealed, and analyzed. The chromatograms were recorded. The peak area (*A*) of each organic solvent was plotted as the vertical axis, and the mass concentration (*ρ*, mg L^−1^) was plotted as the horizontal axis. Linear regression was performed using the least squares method, and the results are shown in Table 1. The results indicated that the eight organic solvents exhibited good linearity within the tested mass concentration range.

##### 2.2.2.4 Reproducibility test

Since the sample did not contain all eight solvents, the reference substances were added for measurement. Six aliquots of the mixed reference solution were precisely weighed, and 5.0 mL of each reference solution was added to a headspace vial, sealed, and mixed thoroughly to dissolve. The measurements were performed following the method outline the chromatograms were recorded. The peak areas of each solvent were measured, and the residual amounts were calculated. The reproducibility of the method was evaluated based on the RSD of the six residual measurements. The results showed that the RSD of the residual amounts for MeOH, DMF, EA, IPAC, TEA, THF, DMF, and *n*-heptane were all less than 5%, indicating that the method has good reproducibility.

##### 2.2.2.5 Spiking recovery test

A 1 g sample of the test substance, which contains a known residual solvent amount, was precisely weighed and placed in a 20 mL headspace vial. Nine aliquots were prepared, and 5.0 mL of reference solution at three different concentrations (50%, 100%, and 150% of each solvent's limit) was added to each vial, with three replicates for each concentration. The nine spiked suvorexant samples were analyzed according to the established method. The total residual solvent amount in each sample was measured, and the background solvent residuals (*e.g.*, methanol) in the test substance were subtracted (the solvent concentrations were calculated using external calibration based on peak area). The recovery rate was calculated based on the added amount, and the results are shown in.

##### 2.2.2.6 Durability test

The suvorexant test solution was analyzed to evaluate its durability under different injection port temperatures, detector temperatures, flow rates, and headspace times. The experimental results are shown in Table 4.

#### 2.2.3 Sample analysis

Table 3 shows that three different production batches of suvorexant Active Pharmaceutical Ingredient (API) were selected for analysis, labeled as Sample A (Batch No. 20240905), Sample B (Batch No. 20240910), and Sample C (Batch No. 20240415). The residual solvents IPAC, *n*-heptane, DMF, TEA, THF, EA, DCM, and MeOH in the three batches were measured using the headspace gas chromatography (HS-GC) method established above.

### 2.3 Discussion of residual solvents

#### 2.3.1 Selection of residual solvents for determination

During the synthesis of suvorexant, eight organic solvents were employed, including IPAC, *n*-heptane, DMF, TEA, EA, DCM, MeOH, and THF. Among these, DCM, DMF, and THF are classified as toxic or potentially carcinogenic solvents. Even at low residual levels, prolonged exposure may lead to bioaccumulation, posing significant risks to patient safety. Therefore, accurate and sensitive quantification of these residual solvents is essential for ensuring the quality and safety of the API.

### 2.4 Selection of determination conditions for residual solvents

#### 2.4.1 Selection of chromatographic columns

In this study, an Agilent DB-624 capillary column (30 m × 0.53 mm, 3 μm) was employed for the determination of eight residual organic solvents in suvorexant. DB-624 is a medium-polarity column composed of 6% cyanopropylphenyl and 94% dimethylpolysiloxane, offering excellent separation efficiency and selectivity, particularly suitable for residual solvent analysis. The selected solvents span a wide polarity range, including non-polar (*e.g.*, *n*-heptane), weakly polar (*e.g.*, triethylamine), moderately polar (*e.g.*, ethyl acetate, dichloromethane, tetrahydrofuran), and strongly polar (*e.g.*, DMF and methanol) compounds. The intermediate polarity of the DB-624 column facilitates efficient separation of both polar and non-polar solvents, with favorable retention behavior, resolution, and peak symmetry. Moreover, the column exhibits strong resistance to contamination and low bleed characteristics, making it highly compatible with high-sensitivity FID.^27^ This minimizes baseline drift and enhances quantification precision. Therefore, the DB-624 column was selected as the optimal stationary phase to ensure the reliability, reproducibility, and accuracy of residual solvent analysis in suvorexant.

#### 2.4.2 Selection of detectors and carrier gases

This analytical method employed a FID, owing to its high sensitivity for detecting carbon-containing compounds, especially in matrices with low oxygen content. Nitrogen was selected as the carrier gas in the HS-GC system for several key reasons. From a cost perspective, nitrogen is widely available, easily generated, and significantly more economical than helium, a noble gas commonly used in GC, thereby reducing long-term operational costs and ensuring stable carrier gas supply. In terms of safety, nitrogen is chemically inert and non-reactive with sample components, minimizing the risk of analyte degradation or interference. Its non-flammable nature further enhances laboratory safety by eliminating the potential for combustion or explosion.

In terms of system compatibility, nitrogen demonstrates excellent suitability for use with FID by providing a stable combustion environment. Through appropriate method optimization, nitrogen meets the sensitivity and resolution requirements of the analysis. Furthermore, its compatibility with the selected DB-624 capillary column ensures effective chromatographic separation without compromising column performance or longevity. Given the nature of the target analytes and the precision requirements of this study, nitrogen proved to be a practical choice, balancing analytical accuracy, cost-effectiveness, and operational safety.

#### 2.4.3 Selection of headspace diluent

In this study, water, acetonitrile (ACN), and dimethyl sulfoxide (DMSO) were evaluated as potential diluents for assessing their influence on the detection of target residual solvents. Experimental results showed that suvorexant exhibited limited solubility in water, leading to reduced responses for certain analytes under HS-GC conditions. Although ACN demonstrated moderate solubility and general compatibility, minor interferences were observed within the retention time window of several analytes. In contrast, DMSO provided superior baseline stability and exhibited no interfering peaks within the retention times of the eight target organic solvents.

While DMSO may generate trace amounts of dimethyl sulfide (DMS) during thermal equilibration, under the optimized chromatographic conditions employed in this study, the DMS peak was well resolved from all target analytes and did not interfere with quantitative analysis. Consequently, DMSO was selected as the diluent of choice for the quantification of residual solvents in suvorexant by HS-GC.

#### 2.4.4 Analytical challenges arising from solvent properties

A key challenge in the experiment was the close retention times of isopropyl acetate (IPAC) and triethylamine (TEA). This phenomenon could be primarily attributed to three factors. Firstly, both compounds relied mainly on hydrophobic interactions (van der Waals forces) for retention on the DB-624 column, which is a medium-polar stationary phase. IPAC, as a medium-polar ester, and TEA, despite containing nitrogen atoms but with overall low polarity, exhibited similar interaction patterns with the stationary phase. Secondly, their boiling points were close (88.6 °C *vs.* 89.5 °C), resulting in similar vapor pressures and volatility under the same chromatographic conditions. Thirdly, at lower heating rates, their elution rates were comparable, leading to peak overlap.^27^

Increasing the heating rate proved beneficial. It accelerated the elution of high-boiling components, widening the gap in retention times. Additionally, it reduced the residence time of compounds on the stationary phase, weakening the stationary phase–solute interactions (especially the weak polar interactions like hydrogen bonding or dipole–dipole interactions between the polar groups of TEA and the stationary phase), thus avoiding peak tailing. Moreover, a higher heating rate diminished the longitudinal diffusion effect of peaks, narrowing the peaks and reducing co-elution, thereby improving resolution.

Different heating rates were investigated. When heating at 15 °C min^−1^ to 90 °C, the resolution of IPAC and TEA was less than 1.5. In contrast, a heating rate of 20 °C min^−1^ to 90 °C yielded better resolution. Therefore, the latter heating rate was ultimately adopted in the experiment.

#### 2.4.5 Correlation between physicochemical properties of solvents and detection performance

Table 2 summarizes the recovery and RSD of eight residual solvents across concentrations, revealing that analytical performance is governed by synergies between experimental conditions and solvent properties (boiling point, polarity, thermal stability). Methanol showed excellent recovery (103.1%) and precision (RSD = 1.04%) due to high polarity and low boiling point (64.7 °C) enabling rapid gas–liquid equilibrium. DMF also performed well (recovery = 101.1%, RSD = 0.18%), attributed to high boiling point (153 °C) for prolonged vaporization, strong dipole moment (3.8 D) enhancing stationary phase interaction, and minimized volatilization loss.

**Table 2:** Results of recovery test (*n* = 9)^a^

Compound	Average recovery/% ( *n* = 3)			Average recovery/%	RSD/%
	50%	100%	150%		
MeOH	103.4	102.2	103.7	99.05	2.51
DCM	99.2	99.2	100.9	98.47	1.81
EA	96.1	96.0	97.3	98.27	1.49
THF	92.9	92.4	92.4	97.76	1.35
IPAC	98.2	98.9	99.1	103.63	3.39
TEA	85.0	84.2	84.9	98.12	3.28
*n*-Heptane	120.4	118.8	121.3	97.18	2.56
DMF	101.3	101.0	101.0	98.26	3.77

a (1) MeOH (methanol) (2) DCM (dichloromethane) (3) EA (ethyl acetate) (4) THF (tetrahydrofuran) (5) IPAC (isopropyl acetate) (6) TEA (triethylamine) (7) *n*-heptane (8) DMF (*N*,*N*-dimethylformamide).

**Table 3:** Residual solvent contents of three batch test samples

Sample batch	TEA (%)	*n*-Hep (%)
20240415	0.001	0.004
20240905	0.001	0.004
20240910	0.001	0.005

Non-polar *n*-heptane exhibited anomalously high recovery (120.20%, RSD = 1.06%), potentially from detector overresponse to low-polarity analytes (log *P* = 4.0) or matrix interference, mitigable *via* detector linear range adjustment or internal standards (*e.g.*, decane). TEA had the lowest recovery (84.75%, RSD = 0.47%), likely due to thermal degradation at injection temperatures >215 °C (boiling point = 89 °C), suggesting gradient heating to reduce thermal exposure. THF showed moderate losses (92.63%, RSD = 0.26%) from azeotropy with water (66 °C), alleviable by optimizing headspace temperature to 85 °C. Isopropyl acetate (98.79%, RSD = 0.45%) had slight deviation from hydrophobicity (log *P* = 1.7) slowing partitioning, improved by 32 min equilibration.

These findings highlight dynamic interactions between solvent properties and analytical parameters. DMF's “high boiling-high stability” profile requires co-adjusting temperature and carrier flow to balance vaporization and trapping. *n*-Heptane's exaggerated response indicates potential bias in low-polarity solvent detection, valuable for complex matrices. Further research should explore solvent-stationary phase adsorption kinetics and QSAR-based models to optimize detection parameters.

Table 4 shows carrier gas flow, temperature, and equilibration time jointly affect recovery and reproducibility. Lower flow (1.8 mL min^−1^) improved DMF recovery but increased RSD, while higher flow (2.2 mL min^−1^) benefited high-boiling solvent mass transfer and reduced RSD. Temperature effects varied with volatility: 95 °C headspace reduced DMF recovery; 225 °C injection degraded TEA; 285 °C detector enhanced low-boiling solvent response but increased baseline noise. Prolonged equilibration (32 min) reduced EA's RSD; *n*-heptane's slow partitioning at 28 min reflected polarity-dependent equilibrium.

**Table 4:** Results of durability investigation experiment

Factor	Condition	MeOH recovery/RSD (%)	DCM recovery/RSD (%)	EA recovery/RSD (%)	THF recovery/RSD (%)	IPAC recovery/RSD (%)	TEA recovery/RSD (%)	*n*-Hept recovery/RSD (%)	DMF recovery/RSD (%)
Carrier gas flow rate	1.8 ml min^−1^	103.9/3.8	104.2/2.38	105.3/2.26	105.0/1.86	106.0/3.05	106.0/3.48	106.6/4.44	106.5/3.47
	2.2 ml min^−1^	101.3/1.60	100.4/1.91	101.2/2.47	101.9/2.39	102.0/3.08	102.7/4.6	104.2/5.09	100.6/3.92
Headspace temperature	85 °C	102.7/0.86	101.3/1.45	102.0/1.70	102.4/1.85	103.0/1.62	103.1/2.84	105.5/2.87	102.7/1.73
	95 °C	101.1/0.79	100.1/1.88	100.8/2.62	101.5/2.79	101.5/2.52	101.8/4.45	103.6/5.86	97.9/3.93
Injector temperature	215 °C	100.1/2.25	100.4/2.96	100.4/3.37	100.5/3.19	100.0/3.82	100.3/4.57	100.1/5.35	100.5/2.81
	225 °C	98.5/2.22	97.6/2.10	97.3/2.30	97.3/1.87	97.2/3.41	96.9/3.07	96.3/3.40	99.4/4.05
Detector temperature	275 °C	100.0/1.55	98.361.40	97.6/1.52	97.3/1.48	97.8/1.60	93.4/1.94	92.5/2.05	103.6/4.76
	285 °C	104.2/2.32	104.8/1.63	105.7/1.59	104.9/1.31	106.4/2.44	106.3/1.81	106.8/2.62	108.0/4.39
Headspace equilibration time	28 min	98.6/2.68	96.8/1.58	96.2/1.98	97.5/1.35	96.9/2.27	95.7/1.86	96.2/1.80	97.3/4.91
	32 min	100.3/1.89	100.1/2.96	100.8/3.57	100.7/3.72	100.5/3.12	101.2/4.99	101.6/5.08	100.9/3.2

In conclusion, parameters should align with solvent properties to balance recovery and precision. A generalized setting (2.2 mL min^−1^ flow, 85 °C headspace, 32-min equilibration) is recommended. Thermally labile solvents (*e.g.*, TEA) require injection temperatures ≤215 °C; high-boiling solvents (*e.g.*, DMF) benefit from 285 °C detector temperature with optimized flow. Consistent RSDs <5% confirm robustness for diverse residual solvent analyses. Future work should clarify solvent-stationary phase interactions and develop predictive models for automated optimization.

## 3. Conclusion

In this study, a novel synthetic process for suvorexant based on chiral starting materials was developed and optimized. We can see that most of the synthetic routes in the supplement data have relatively low yields and involve complicated steps. Therefore, the purpose of this route is to reduce complicated steps, lower production costs, and develop a new synthetic route with high yield and high efficiency. Compared with traditional routes, this process eliminates the need for chiral resolution and avoids the use of highly toxic intermediates. This strategy achieves an overall yield of 65% for the final six steps and boasts advantages such as low cost, compliance with green chemistry concepts, and strong industrial operability. Meanwhile, a headspace gas chromatography-flame ionization detection (HS-GC-FID) method was established for the quantitative analysis of 8 residual solvents. This method exhibits stable performance, conforms to the ICH Q3C guidelines, and shows reliable repeatability under multiple sets of validation conditions. Moreover, this analytical method can guide the synthetic process in controlling residual solvents. The analysis results of three batches of active pharmaceutical ingredient (API) samples revealed only trace amounts of triethylamine and *n*-heptane, indicating that the optimized process can effectively control residual solvents. By integrating process optimization with solvent residue control, this study provides an important reference for the research and development of suvorexant and other small-molecule drugs, facilitating their subsequent industrial applications. Future research can focus on exploring green solvent alternatives and developing automated synthesis workflows to promote the sustainable production of this drug.

## 4. Experimental section

### 4.1 General methods

#### 4.1.1 Materials and instruments for synthesis experiments

^1^H and ^13^C NMR spectra were acquired on Bruker AVANCE NEO 600 MHz NMR spectrometer to characterize the chemical structures. For detailed information, refer to the SI. High-Performance Liquid Chromatography (HPLC) was conducted with an instrument from Thermo (USA).

The following reagents were provided by Anhui Zesheng Technology Co., Ltd: 5-methyl-2-(2*H*-1,2,3-triazol-2-yl)benzoic acid, 2,5-dichlorobenzoxazole, *N*-methylglycine ethyl ester, 1-hydroxybenzotriazole, sodium methoxide, 1-(3-dimet-hylaminopropyl)-3-ethylcarbodiimide hydrochloride, (*R*)-3-(Boc-amino)butyric acid, lithium aluminum hydride, and oxalyl chloride. The following reagents were supplied by China National Pharmaceutical G-roup Corporation (Sinopharm Group Chemical Reagent Co., Ltd): IPAC, THF, DMF, EA, *n*-heptane, 4.0 mol per L hydrochloric acid-ethyl acetate solution, sodium hydroxide, anhydrous sodium sulfate, potassium carbonate, triethylamine, anhydrous magnesium sulfate, sodium chloride, anhydrous citric acid, methanol, and DCM.

#### 4.1.2 Materials and instruments for residual solvent method development

An Agilent 7890B gas chromatograph (equipped with a Flame Ionization Detector (FID), ChemStation chromatography workstation, and Agilent 7697A headspace sampler, Agilent Technologies, USA) was used. Dimethyl sulfoxide (DMSO) was used as the solvent (GC-HS grade, batch number E052432, Anheji Chemical Co., Ltd). Methanol was used as the gas chromatography standard (analytical grade, batch number F23N5M201, Fisher Chemical, USA). DCM, EA, THF, IPAC, *n*-heptane, and DMF were all used as gas chromatography standards (analytical grade, batch numbers 20240608, 20240711, 20230120, 20230113, 20240313, 20240509, Sinopharm Group Chemical Reagents Co., Ltd). Suvorexant (batch number 20240910) was obtained from the Institute of Toxicology and Pharmacology, Academy of Military Medical Sciences, Beijing.

### 4.2 Synthetic procedures

#### 4.2.1 Synthesis of ethyl (*R*)-*N*-benzyl-*N*-(3-((*tert*-butoxycarbonyl) amino) butanoyl) glycinate

Dissolve 1000 g of compound 3 (*R*)-3-(Boc-amino)butanoic acid in 4 L of *N*,*N*-dimethylformamide. Subsequently, 950 g of *N*-benzylglycine ethyl ester, 831 g of 1-hydroxybenzotriazole (HOBt), and an appropriate amount of triethylamine were added, with continued stirring to ensure full dissolution. The system was then placed in an ice-water bath and cooled to below 10 °C. Under stirring, 1131 g of 1-ethyl-3-(3-dimethylaminopropyl)carbodiimide hydrochloride (EDCI) was added in three portions, with temperature control to maintain the reaction temperature below 20 °C after each addition. After the final addition, stirring was continued for 4 h, and the temperature was then raised to 30 °C for an additional 2 h. Upon completion of the reaction, the mixture was cooled to room temperature, and 4.8 L of 10% citric acid solution was added slowly to quench the reaction. The organic phase was extracted with 9 L of ethyl acetate, washed once with 5 L of 5% potassium carbonate solution, and then washed with 1 L of saturated sodium chloride solution.

The organic phase was dried over anhydrous sodium sulfate, filtered, and the filtrate was concentrated under reduced pressure, compound 5 yielding 2.5 L of a yellow oily substance (no need for full purification, based on the starting material, with a yield calculated as 100%). ^1^H NMR (600 MHz, DMSO) *δ* 7.40–7.18 (m, 5H), 6.71 (t, *J* = 9.1 Hz, 1H), 4.79–4.54 (m, 2H), 4.33 (dd, *J* = 90.0, 16.8 Hz, 1H), 4.07 (p, *J* = 7.1 Hz, 2H), 3.98 (dd, *J* = 39.1, 16.9 Hz, 1H), 3.90–3.78 (m, 1H), 2.56 (ddd, *J* = 20.6, 15.3, 4.9 Hz, 1H), 2.28 (ddd, *J* = 58.3, 15.2, 8.3 Hz, 1H), 1.36 (d, *J* = 7.8 Hz, 9H), 1.16 (td, *J* = 7.1, 5.0 Hz, 3H), 1.05 (dd, *J* = 12.8, 6.6 Hz, 3H). MS (ESI) *m*/*z* 279.1705 [M + H]^+^.

#### 4.2.2 Synthesis of ethyl (*R*)-*N*-(3-aminobutanoyl)-*N*-benzylglycinate

Compound 5 dissolved in 300 mL of ethyl acetate and cooled to 10 °C in an ice-water bath. Subsequently, 2.5 L of 4 N Hydrogen Chloride in Ethyl Acetate (HCl/EA) was added dropwise under stirring. The reaction mixture was stirred at 10 °C for 1 h, then gradually warmed to 50 °C and stirred for an additional 2 h.

Upon completion of the reaction, 8 L of water was added, and the mixture was thoroughly stirred and subjected to phase separation. The aqueous layer was collected and adjusted to pH 9–10 using 25% potassium carbonate solution, followed by extraction with 6 L of ethyl acetate. The organic phases were combined, dried over anhydrous sodium sulfate, filtered, and concentrated under reduced pressure to afford 1360 g of compound 6 as a yellow oily product. Based on the starting material, this corresponds to a 100% theoretical yield, and the product requires no purification.

#### 4.2.3 Synthesis of (*R*)-4-benzyl-7-methyl-1,4-diazepane-2,5-dione

Dissolve compound 6 in 4.8 L of methanol. Sodium methoxide (288 g) was then added slowly at room temperature, and the reaction mixture was stirred for 4 h. Upon completion, the reaction was quenched with 1.8 L of saturated ammonium chloride solution. The solvent was removed under reduced pressure, and the residue was treated with 1.5 L of saturated potassium carbonate solution, followed by extraction with 2 L of dichloromethane. The organic layer was washed with 500 mL of saturated sodium chloride solution, dried over anhydrous sodium sulfate, filtered, and concentrated under reduced pressure to yield a white solid. The obtained solid was slurried in 900 mL of ethyl acetate under stirring, and then 2.7 L of *n*-heptane was added slowly. The slurry was stirred for an additional 2 h. The resulting white suspension was filtered, and the filter cake was washed with a mixture of ethyl acetate and *n*-heptane (v/v = 1 : 3). The solid was placed in a vacuum oven and dried at 50 °C for 10 hours to obtain 810 g of compound 7. Based on the total mass of compounds 3 to 7, the overall yield was calculated to be 71%.^1^H NMR (600 MHz, DMSO) *δ* 7.70 (s, 1H), 7.37–7.18 (m, 5H), 4.60–4.48 (m, 2H), 4.04 (q, *J* = 17.2 Hz, 2H), 3.67–3.57 (m, 1H), 2.81 (ddd, *J* = 22.8, 14.3, 5.9 Hz, 2H), 1.12 (d, *J* = 6.4 Hz,3H). MS (ESI) *m*/*z* 233.1286 [M + H]^+^.

#### 4.2.4 Synthesis of ethyl (*R*)-*N*-(3-aminobutanoyl)-*N*-benzylglycinate

Dissolve compound 7 in 16 L of tetrahydrofuran (THF). The mixture was stirred until fully dissolved. The flask was purged with nitrogen to replace the air and maintained under a continuous nitrogen flow. Under an ice-water bath, the reaction temperature was kept below 10 °C, and lithium aluminum hydride (LiAlH_4_, 288 g) was added in portions. The reaction mixture was stirred for 4 h, then gradually warmed to 30 °C and stirred for an additional 2 h under nitrogen protection to prevent atmospheric exposure.

After completion of the reaction, the mixture was cooled to below 10 °C. Water (288 mL) was added dropwise over more than 1 h using a pressure-equalizing dropping funnel. This was followed by the slow addition of 288 mL of 15% sodium hydroxide solution, then an additional 148 mL of water and 430 g of magnesium sulfate (MgSO_4_). The reaction mixture was stirred and allowed to warm to room temperature. After confirming cessation of gas evolution, the mixture was filtered under vacuum.

The filtrate was dried over anhydrous sodium sulfate, filtered, and concentrated under reduced pressure to give 694 g of compound 8 as a yellow liquid, with a corresponding yield of 89%. ^1^H NMR (600 MHz, DMSO) *δ* 7.40–7.18 (m, 5H), 6.71 (t, *J* = 9.1 Hz, 1H), 4.79–4.54 (m, 2H), 4.33 (dd, *J* = 90.0, 16.8 Hz, 1H), 4.07 (p, *J* = 7.1 Hz, 2H), 3.98 (dd, *J* = 39.1, 16.9 Hz, 1H), 3.90–3.78 (m, 1H), 2.56 (ddd, *J* = 20.6, 15.3, 4.9 Hz, 1H), 2.28 (ddd, *J* = 58.3, 15.2, 8.3 Hz, 1H), 1.36 (d, *J* = 7.8 Hz, 9H), 1.16 (td, *J* = 7.1, 5.0 Hz, 3H), 1.05 (dd, *J* = 12.8, 6.6 Hz, 3H). MS (ESI) *m*/*z*305.2231 [M + H]^+^.

#### 4.2.5 Synthesis of tert-butyl (*R*)-4-benzyl-7-methyl-1,4-diazepane-1-carboxylate

Compound 8 was dissolved in 2.7 L of methanol. The mixture was stirred until dissolved and then cooled to below 10 °C in an ice-water bath. Di-*tert*-butyl dicarbonate (Boc_2_O, 881 g) was added slowly, followed by the slow addition of triethylamine (409 g). The reaction was stirred at this temperature for 3 h.

Upon completion, the reaction mixture was concentrated under reduced pressure. The residue was quenched with 7 L of 10% aqueous citric acid solution. Extraction was performed with 3 L of ethyl acetate, and the aqueous layer was retained. The pH of the aqueous layer was adjusted to 8–9 using 6 L of 30% sodium hydroxide solution, resulting in the precipitation of a white solid. The mixture was extracted with 3 L of ethyl acetate. The organic phases were combined, washed with 1 L of saturated sodium chloride solution, dried over anhydrous sodium sulfate, filtered, and concentrated under reduced pressure to obtain 925 g of compound 9 as a brown liquid, with a yield of 91%.^1^H NMR (600 MHz, DMSO) *δ* 4.03–3.85 (m, 1H), 3.74–3.43 (m, 1H), 3.29–3.03 (m, 1H), 2.97–2.63 (m, 1H), 2.59–2.20 (m, 1H), 1.98 (qd, *J* = 14.2, 6.7 Hz, 1H), 1.51–1.27 (m, 1H), 1.01 (dd, *J* = 23.6, 6.3 Hz, 1H). MS (ESI) *m*/*z* 159.1127 [M + H]^+^.

#### 4.2.6 Synthesis of tert-butyl (*R*)-7-methyl-1,4-diazepane-1-carboxylate

Compound 9 was dissolved in 5 L of methanol and 90 g of 10% palladium on carbon (Pd/C). The flask was purged with hydrogen gas to replace the internal atmosphere, and the hydrogenation reaction was carried out under a controlled temperature of 30–35 °C with continuous stirring.

Upon completion, the reaction mixture was filtered under vacuum. The filter cake was washed with methanol, and the combined filtrate was dried over anhydrous sodium sulfate. After filtration and concentration under reduced pressure, 640 g of compound 10 was obtained as an oily compound 10, corresponding to a yield of 96%.^1^H NMR (600 MHz, DMSO) *δ* 7.40 (d, *J* = 8.4 Hz, 1H), 7.31 (d, *J* = 1.7 Hz, 1H), 7.01 (dd, *J* = 8.4, 1.6 Hz, 1H), 4.22–3.19 (m, 1H), 2.241.86 (m, 1H), 1.64 (d, *J* = 5.7 Hz, 1H), 1.37 (s, 1H), 1.09 (d, *J* = 6.3 Hz, 1H). MS (ESI) *m*/*z* 366.1580 [M + H]^+^.

#### 4.2.7 Synthesis of tert-butyl (*R*)-4-(5-chlorobenzo[*d*]oxazol-2-yl)-7-methyl-1,4-diazepane-1-carboxylate

Compound 10 was dissolved in 5.9 L of dichloromethane. After complete dissolution, the temperature was maintained below 10 °C. Then, 2,5-dichlorobenzoxazole (517.7 g) was added, followed by the slow addition of triethylamine (308 g). The reaction mixture was allowed to warm to room temperature and stirred for 4 h.

Upon completion, 2 L of DCM was added, and the mixture was washed and extracted with water. The organic phase was subsequently washed with 1.5 L of 10% aqueous citric acid solution and then with 900 mL of saturated sodium chloride solution. The organic phase was dried over magnesium sulfate, filtered, and concentrated under reduced pressure to obtain compound 12 as an oily compound (1137 g) with a yield of 99%. ^1^H NMR (600 MHz, DMSO) *δ* 7.40 (d, *J* = 8.4 Hz, 1H), 7.31 (d, *J* = 1.7 Hz, 1H), 7.01 (dd, *J* = 8.4, 1.6 Hz, 1H), 4.22–3.19 (m, 1H), 2.24–1.86 (m, 1H), 1.64 (d, *J* = 5.7 Hz, 1H), 1.37 (s, 1H), 1.09 (d, *J* = 6.3 Hz, 1H). MS (ESI) *m*/*z* 366.1580 [M + H]^+^.

#### 4.2.8 Synthesis of (R)-5-chloro-2-(5-methyl-1,4-diazepan-1-yl)benzo[*d*]oxazole

Compound 12 was dissolved in 200 mL of ethyl acetate and cooled to 10 °C. Then, 3.5 L of 4 N hydrogen chloride in ethyl acetate was added, and the reaction mixture was stirred at 10 °C for 2.5 h. Upon completion, the precipitate was collected by vacuum filtration and washed with ethyl acetate.

The resulting solid was suspended in 2.4 L of methanol and heated to 75 °C under reflux for 30 min. Afterward, 6.6 L of isopropyl acetate was added, the stirring rate was reduced, and the mixture was allowed to cool to room temperature while stirring for an additional 2 h.

Subsequently, 3 L of dichloromethane and 2 L 12% sodium hydroxide solution were added, and the pH was adjusted to 11–13. The mixture was stirred for 10 min and extracted with dichloromethane. The organic phase was dried over magnesium sulfate, filtered, and concentrated under reduced pressure to obtain 707 g of *R*-13 as a pale yellow oil, yield: 96%.^1^H NMR (600 MHz, DMSO) *δ* 7.38 (d, *J* = 8.4 Hz, 1H), 7.29 (d, *J* = 2.1 Hz, 1H), 6.98 (dd, *J* = 8.4, 2.2 Hz, 1H), 3.85 (ddd, *J* = 14.3, 5.6, 3.9 Hz, 1H), 3.67 (dt, *J* = 13.3, 3.5 Hz, 1H), 3.59–3.17 (m, 1H), 3.11 (ddd, *J* = 14.2, 4.2, 3.4 Hz, 1H), 2.79 (ddd, *J* = 14.3, 10.7, 3.8 Hz, 1H), 2.72–2.55 (m, 1H), 2.22 (s, 1H), 1.85 (ddt, *J* = 14.0, 6.1, 3.1 Hz, 1H), 1.42 (dtd, *J* = 13.9, 10.0, 3.7 Hz, 1H), 1.01 (d, *J* = 6.4 Hz, 1H). MS (ESI) *m*/*z* 266.1057 [M + H]^+^.

#### 4.2.9 Synthesis of suvorexant

Compound *R*-13 was dissolved in IPAC (3.3 L) and DMF (55 mL) at 25 °C. Oxalyl chloride (394.7 g) was added dropwise, and the reaction mixture was stirred for 1 h. After the reaction, the solvent was evaporated under reduced pressure, and the residue was redissolved in isopropyl acetate (3.3 L) for subsequent use.

(*R*)-5-Chloro-2-(5-methyl-1,4-diazepan-1-yl)benzo[*d*]oxazole was dissolved in isopropyl acetate (10.3 L) and cooled to below 20 °C. Using a constant-pressure dropping funnel, the prepared acyl chloride solution was added slowly to the stirred solution. Triethylamine (524.2 g) was then added, and the mixture was stirred for 3 h.

Afterward, 4.5 L of purified water was added. The temperature was raised to 40–45 °C, and stirring continued until the mixture became clear. The organic phase was separated and washed with saturated sodium chloride solution, dried over magnesium sulfate, filtered, and concentrated under reduced pressure.

The crude product was dissolved in isopropyl acetate (1.2 L) at 80 °C until fully dissolved. The solution was cooled to 65 °C, and suvorexant seed crystals (500 mg) were added. After stirring for 1 h, the temperature was further reduced to 50 °C, and *n*-heptane (9 L) was added dropwise. The suspension was stirred at 45 °C for 5 h. The resulting solid was collected by vacuum filtration, washed with *n*-heptane, and dried in a vacuum oven at 70 °C for 10 h to afford suvorexant 836 g, with a yield of 87%.

^1^H NMR (600 MHz, DMSO-d_6_) *δ* ppm 8.05–7.88 (m, 2H), 7.82–7.78 (m, 1H), 7.42–7.25 (m, 2H), 7.06–7.00 (m, 1H), 4.29–4.06 (m, 1H), 4.01–3.72 (m, 2H), 3.66–3.49 (m, 2H), 2.10 (s, 3H), 2.06–2.01 (m, 1H), 1.78–1.50 (m, 2H), 1.14–1.13 (d, 3H); ^13^C NMR (151 MHz, DMSO) *δ* 168.87 (s), 168.51 (s), 168.07 (s), 163.33 (d, *J* = 19.2 Hz), 147.78 (d, *J* = 10.3 Hz), 145.64–145.25 (m), 138.60 (d, *J* = 22.1 Hz), 136.84–136.29 (m), 134.09 (s), 133.68 (d, *J* = 11.2 Hz), 130.91 (s), 130.75 (d, *J* = 4.8 Hz), 130.57 (s), 129.85 (s), 129.67 (s), 128.81 (d, *J* = 13.2 Hz), 128.59 (s), 128.37 (s), 123.01 (s), 122.82 (s), 122.57 (s), 120.19 (d, *J* = 11.8 Hz), 115.69 (d, *J* = 9.1 Hz), 110.32 (s), 52.38 (s), 51.80 (s), 48.27 (d, *J* = 11.8 Hz), 47.04 (s), 45.08 (s), 44.15 (s), 43.68 (d, *J* = 15.3 Hz), 35.58 (s), 33.51 (s), 20.88 (s), 20.74 (s), 19.70 (s), 18.04 (s), 17.89 (s), 16.76 (s). MS (ESI) *m*/*z* 451.17 [M + H]^+^.

### 4.3 Determination of residual solvents: method development and sample analysis

#### 4.3.1 Chromatographic conditions

The analysis was performed using a DB-624 capillary column (30 m × 0.53 mm, 3 μm film thickness). A temperature-programmed method was employed: the initial oven temperature was set at 55 °C and held for 3 min, then ramped to 90 °C at 20 °C min^−1^ and held for 5 min, followed by a ramp to 220 °C at 25 °C min^−1^, and held for an additional 3 min. The injector temperature was maintained at 220 °C, and the detector temperature at 280 °C. Nitrogen was used as the carrier gas at a flow rate of 2 mL min^−1^ with a split ratio of 5 : 1.

Headspace sampling was employed with the following conditions: headspace equilibration temperature of 90 °C, loop temperature of 100 °C, and transfer line temperature of 110 °C. The pressure equilibration time was set to 30 min. A 20 mL headspace vial containing 5 mL of sample solution was used for the analysis.

#### 4.3.2 Preparation of solutions

##### 4.3.2.1 Blank solvent

Dimethyl sulfoxide (DMSO) was used as the blank solvent. Individual standard solutions: accurately weighed amounts of reference standards 150 mg of methanol, 60 mg of DCM, 250 mg of EA, 250 mg of IPAC, 250 mg of triethylamine, 72 mg of THF, 88 mg of DMF, and 50 mg of *n*-heptane were separately transferred into appropriate volumetric flasks. Methanol, ethyl acetate, isopropyl acetate, and triethylamine were dissolved and diluted to volume in 25 mL flasks using DMSO. Dichloromethane, tetrahydrofuran, *N*,*N*-dimethylformamide, and *n*-heptane were prepared similarly in 50 mL flasks. Each solution was mixed well by shaking and used as individual standard stock solutions.

##### 4.3.2.2 Mixed standard solution

Precisely 5 mL of each individual standard solution was transferred into a 50 mL volumetric flask and diluted to volume with DMSO. The solution was thoroughly mixed for use in the analysis.

##### 4.3.2.3 Test sample solution

Approximately 1 g of the sample was accurately weighed and transferred into a 20 mL headspace vial. Then, 5 mL of the mixed standard solution was added precisely, and the vial was sealed for analysis.

## Author contributions

Chenshuo Jia: conceptualization, methodology, validation, investigation, writing – original draft. Zixing Yu and Yuanyuan Liu: methodology, validation, investigation, writing – original draft, writing – review & editing. Weiguo Shi, Qiao Wang and Aiping Zheng: investigation, writing – review & editing, resources, supervision.

## Conflicts of interest

The authors declare that they have no known competing financial interests or personal relationships that could have appeared to influence the work reported in this paper.

## Supplementary Material

RA-015-D5RA06779K-s001

RA-015-D5RA06779K-s002

## Data Availability

All data generated or analyzed during this study are included in this published article and its supplementary information (SI). Supplementary information: comprise complete characterization spectra of Suvorexant and key intermediates synthesized in this study. Specifically, they include the original ^1^H NMR (Proton Nuclear Magnetic Resonance) and ^13^C NMR (Carbon-13 Nuclear Magnetic Resonance) spectra images of all target compounds and intermediates, which are used to verify the structural accuracy of the compounds. Supplement data: represent a literature review summary of Suvorexant synthetic routes. They systematically sort out the reported synthetic strategies, key reaction steps and route optimization directions related to Suvorexant, providing literature basis for the design and selection of the synthetic route in this paper. See DOI: https://doi.org/10.1039/d5ra06779k.
