# Supplementary material for: Optimized synthesis of suvorexant and determination of eight residual solvents by headspace gas chromatography
Source: RSC Adv. 2025 Oct 30;15(49):41597–607. doi: 10.1039/d5ra06779k (PMC12573263; doi:10.1039/d5ra06779k)
Supplement: RA-015-D5RA06779K-s001 [file RA-015-D5RA06779K-s001.pdf]

Supplementary Information (SI) for RSC Advances.
This journal is © The Royal Society of Chemistry 2025

Supplementary Materials for:

Optimized Synthesis of Suvorexant and Determination of Eight Residual Solvents by Headspace Gas Chromatography

Supplementary materials for NMR spectra of the compounds:

^{1}H NMR

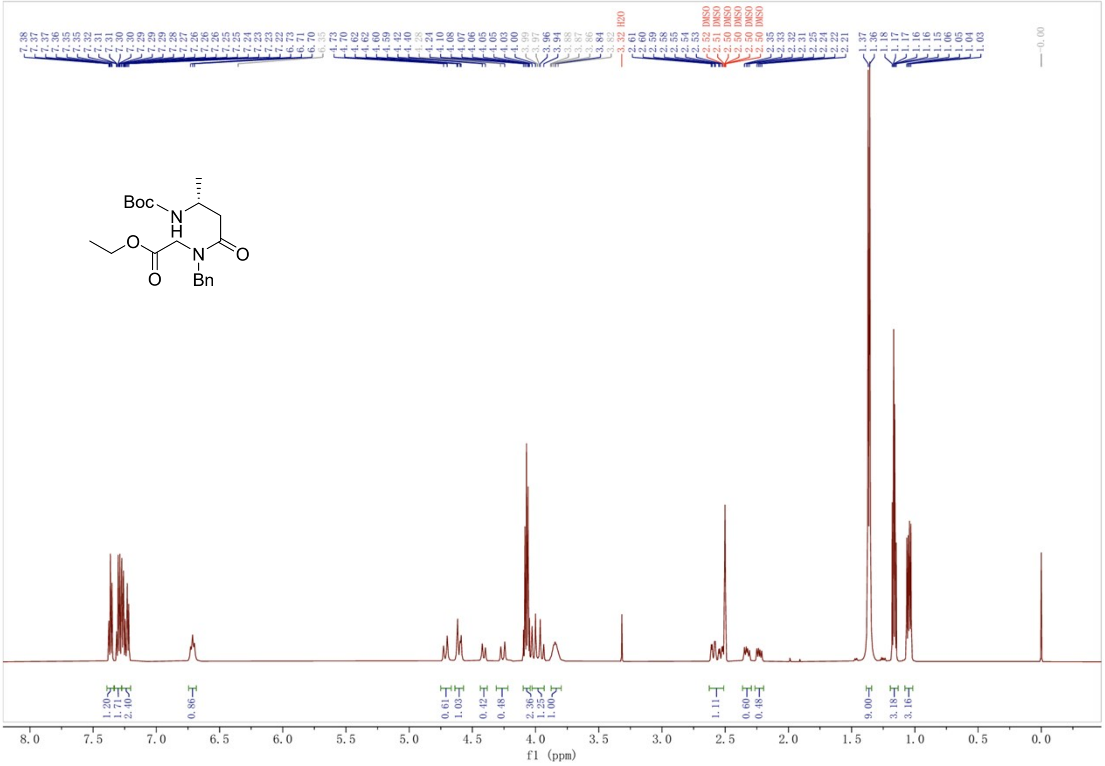

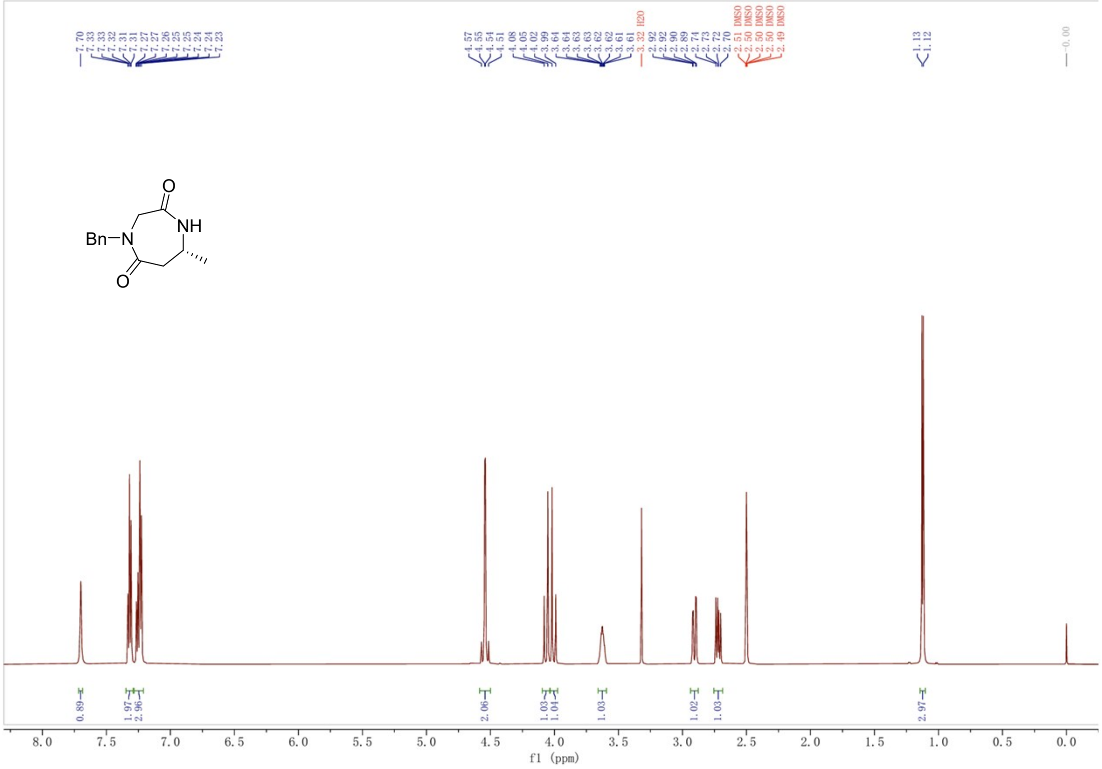

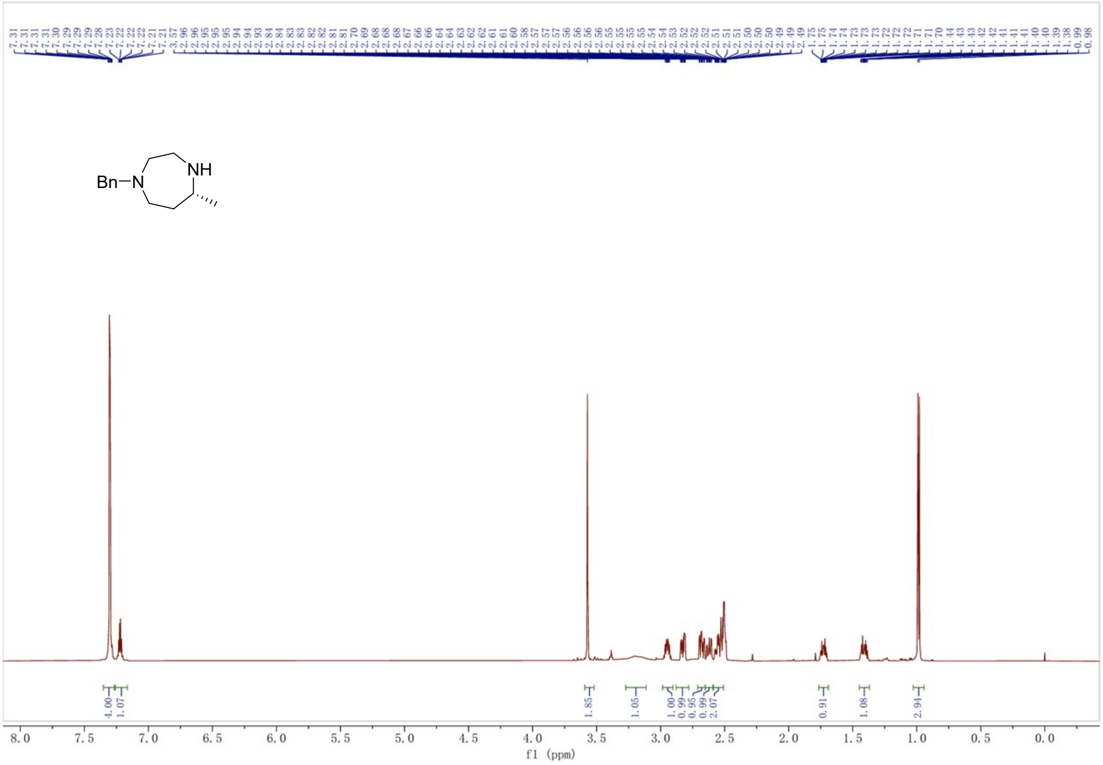
Bn
N
NH
f1 (ppm)

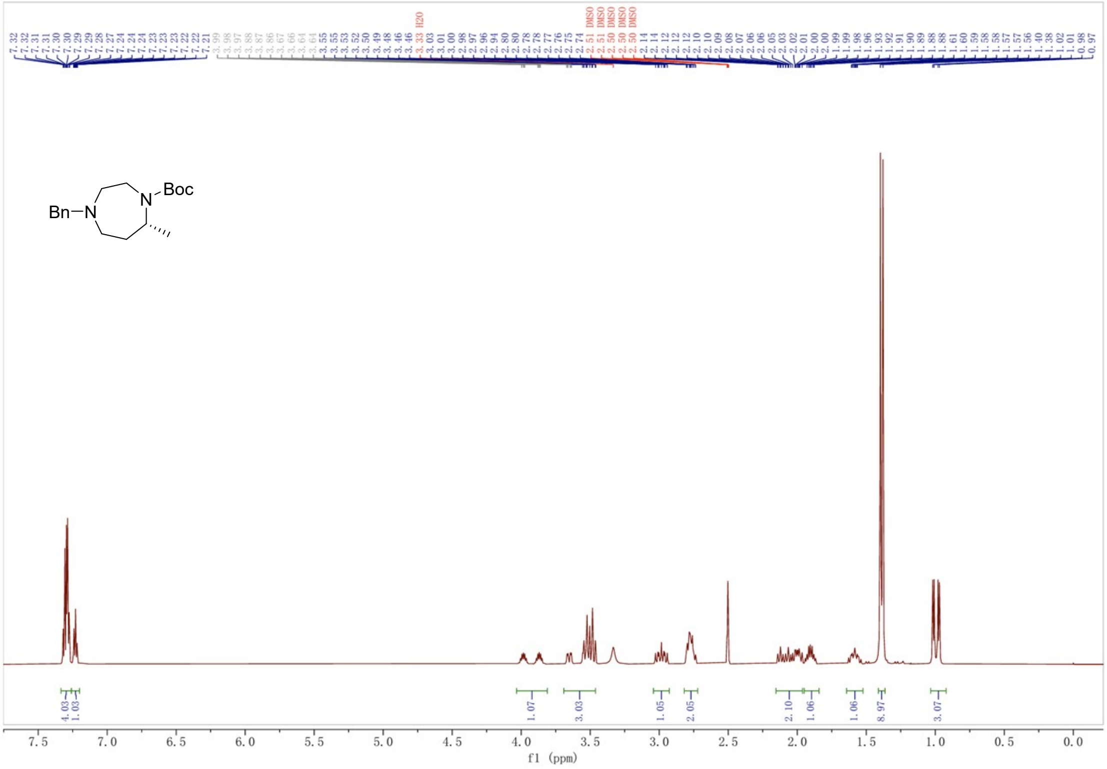
Bn
N
N
Boc
H2O
DMSO
f1 (ppm)

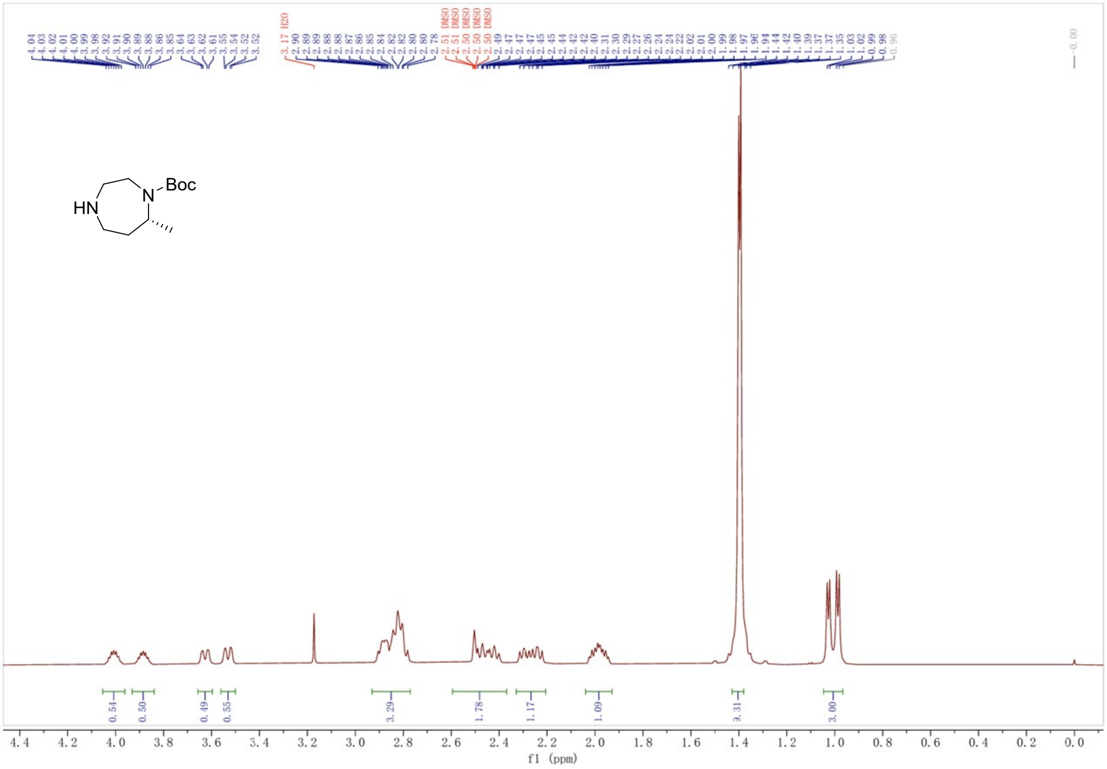
HN
N
Boc
f1 (ppm)

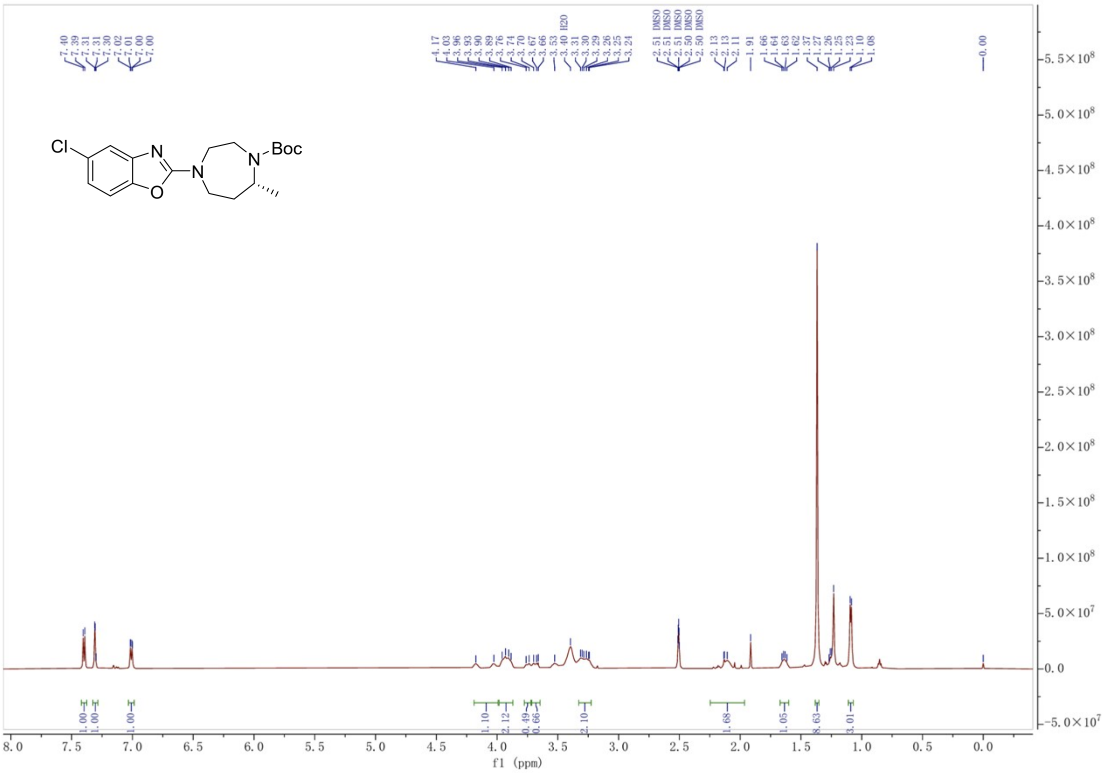
Cl
N
O
N
N
Boc
f1 (ppm)

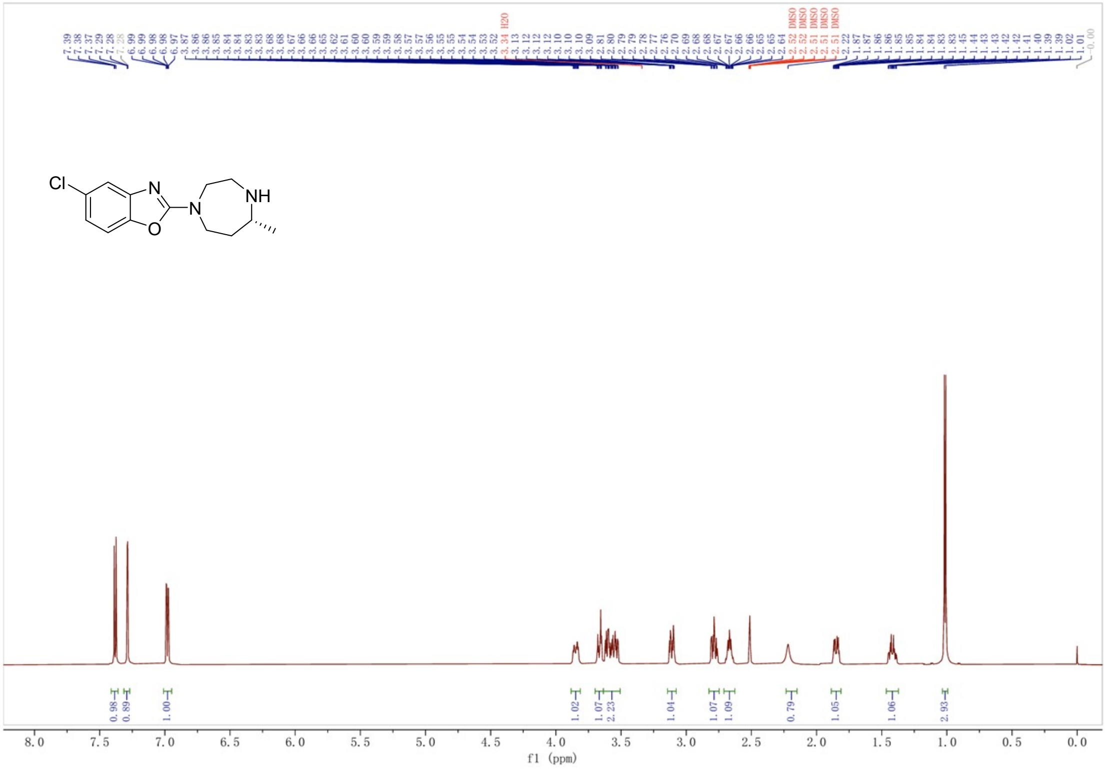
f1 (ppm)

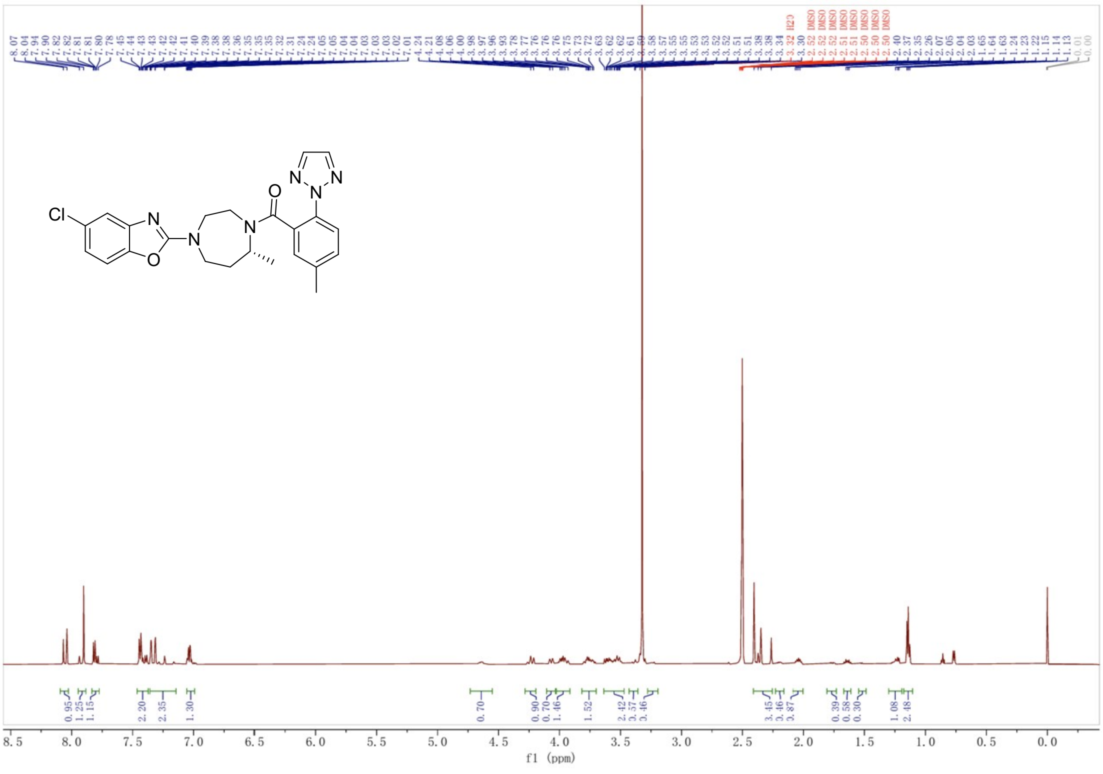
f1 (ppm)

13C NMR

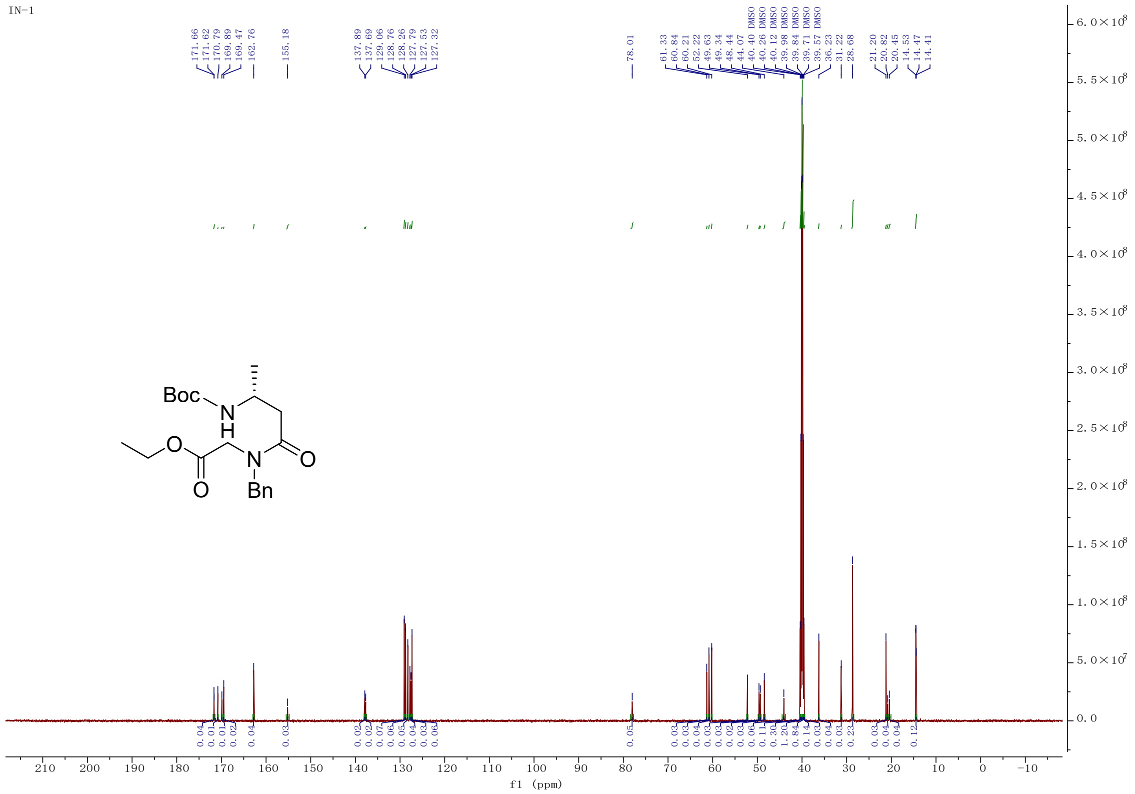
IN-1
171.66
171.62
170.79
169.89
169.47
162.76
155.18
137.89
137.69
129.06
128.76
128.26
127.79
127.53
127.32
78.01
61.33
60.84
60.21
52.22
49.63
49.34
48.44
44.07
40.40 DMSO
40.26 DMSO
40.12 DMSO
39.98 DMSO
39.84 DMSO
39.71 DMSO
39.57 DMSO
36.23
31.22
28.68
21.20
20.82
20.45
14.53
14.47
14.41
Boc
N
H
O
N
O
O
Bn
f1 (ppm)

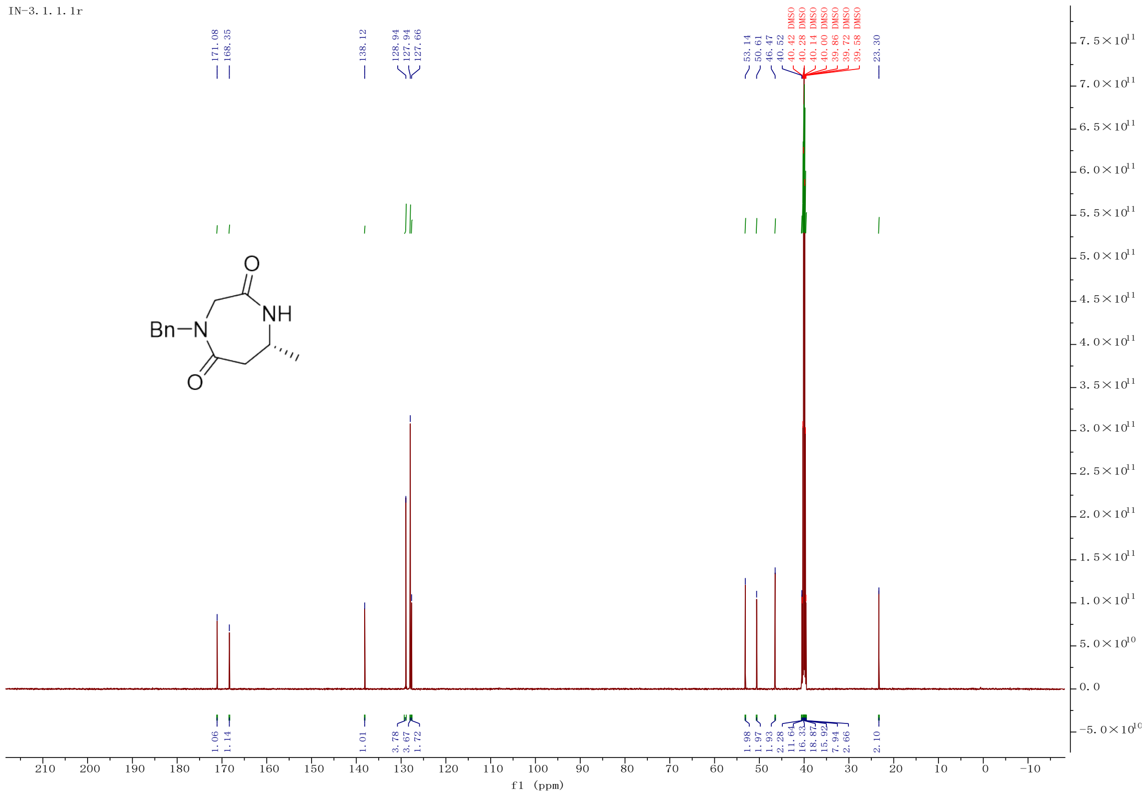
IN-3.1.1.1r
171.08
168.35
138.12
128.94
127.94
127.66
53.14
50.61
46.47
40.52
40.42 DMSO
40.28 DMSO
40.14 DMSO
40.00 DMSO
39.86 DMSO
39.72 DMSO
39.58 DMSO
23.30
O
NH
Bn–N
O
f1 (ppm)

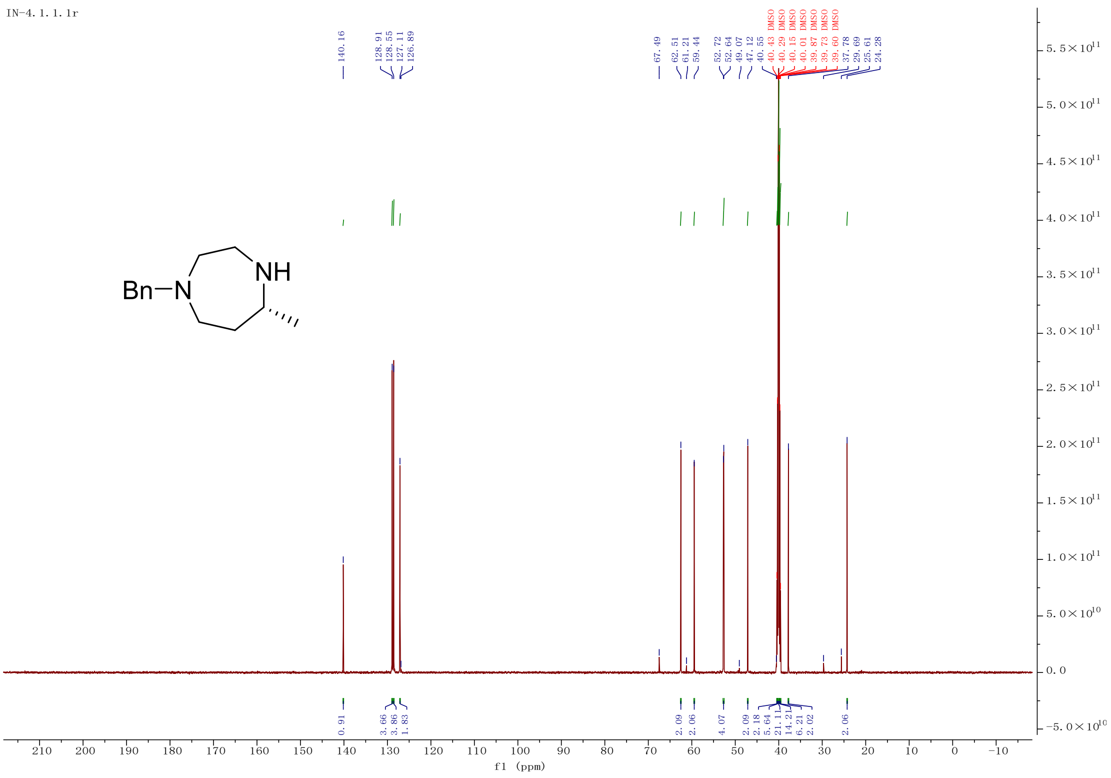
IN-4.1.1.1r
Bn−N
NH
f1 (ppm)

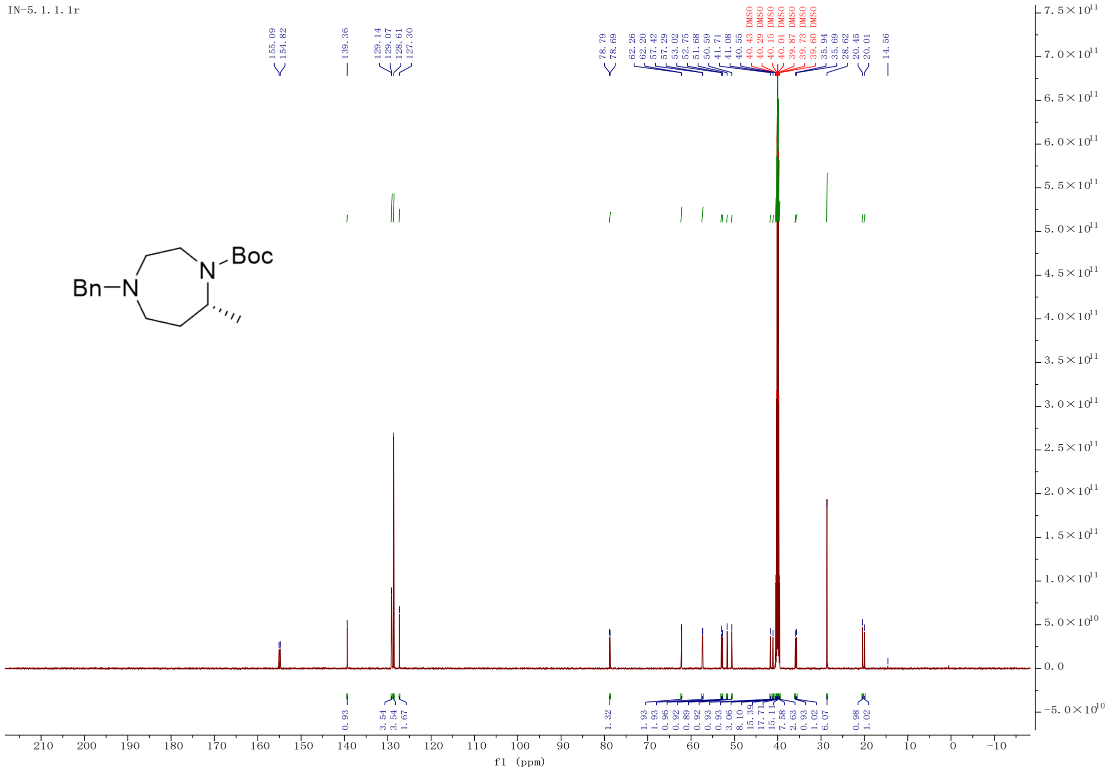
IN-5.1.1.1r
Bn−N
N
Boc
f1 (ppm)

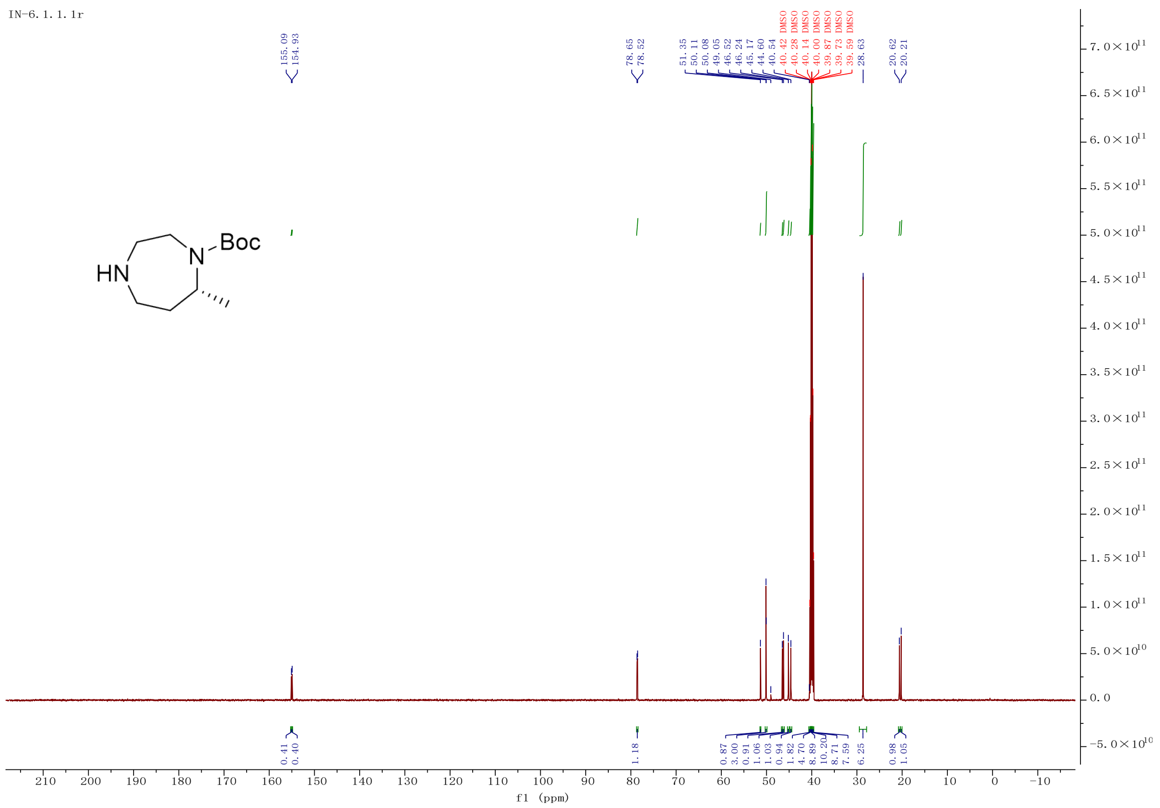
IN-6.1.1.1r
155.09
154.93
78.65
78.52
51.35
50.11
50.08
49.05
46.52
46.24
45.17
44.60
40.54
40.42 DMSO
40.28 DMSO
40.14 DMSO
40.00 DMSO
39.87 DMSO
39.73 DMSO
39.59 DMSO
28.63
20.62
20.21
HN
N
Boc
f1 (ppm)

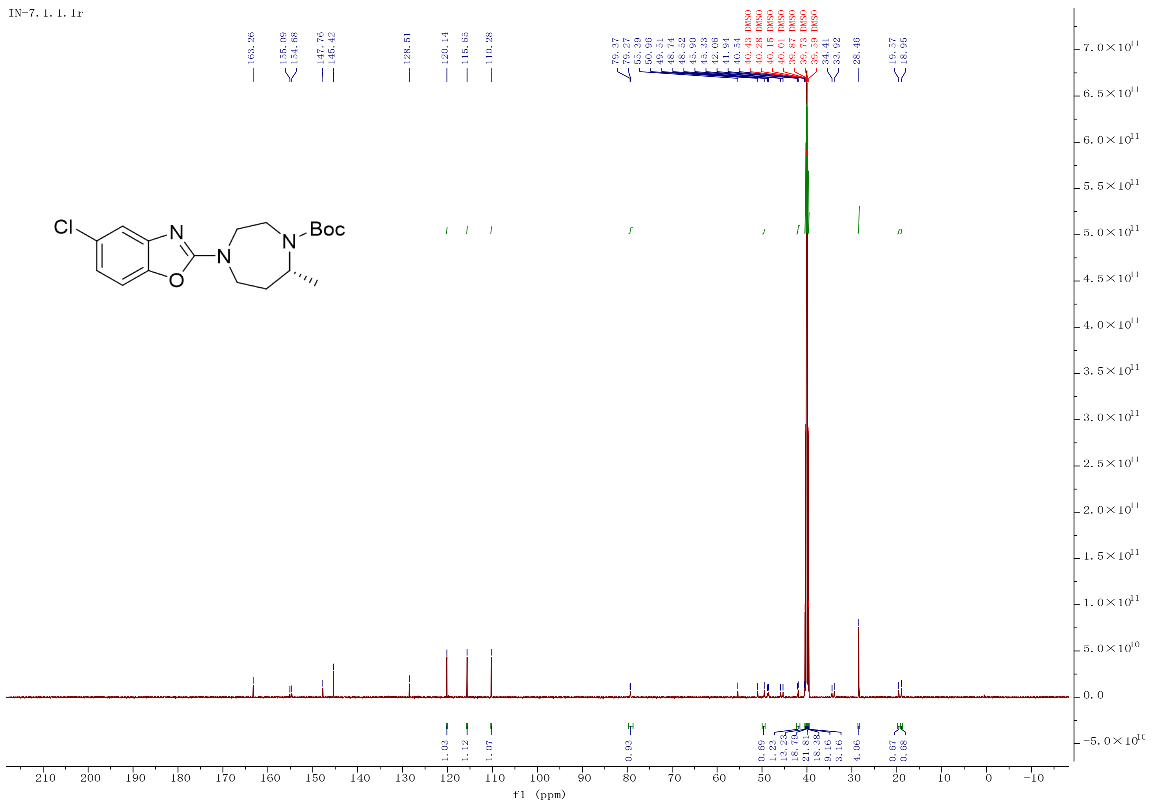
IN-7.1.1.1r
163.26
155.09
154.68
147.76
145.42
128.51
120.14
115.65
110.28
79.37
79.27
55.39
50.96
49.51
48.74
48.52
45.90
45.33
42.06
41.94
40.54
40.43 DMSO
40.28 DMSO
40.15 DMSO
40.01 DMSO
39.87 DMSO
39.73 DMSO
39.59 DMSO
34.41
33.92
28.46
19.57
18.95
Cl
N
O
N
N
Boc
f1 (ppm)

IN-8.1.1.1r

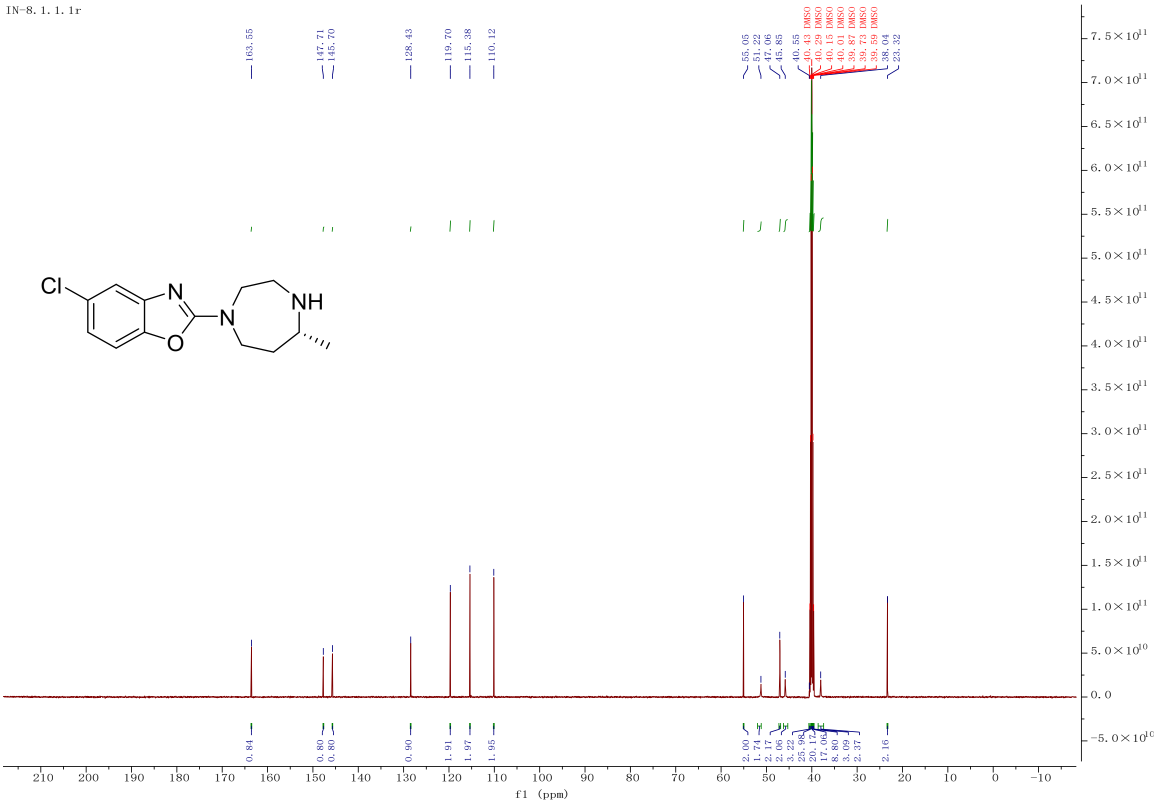

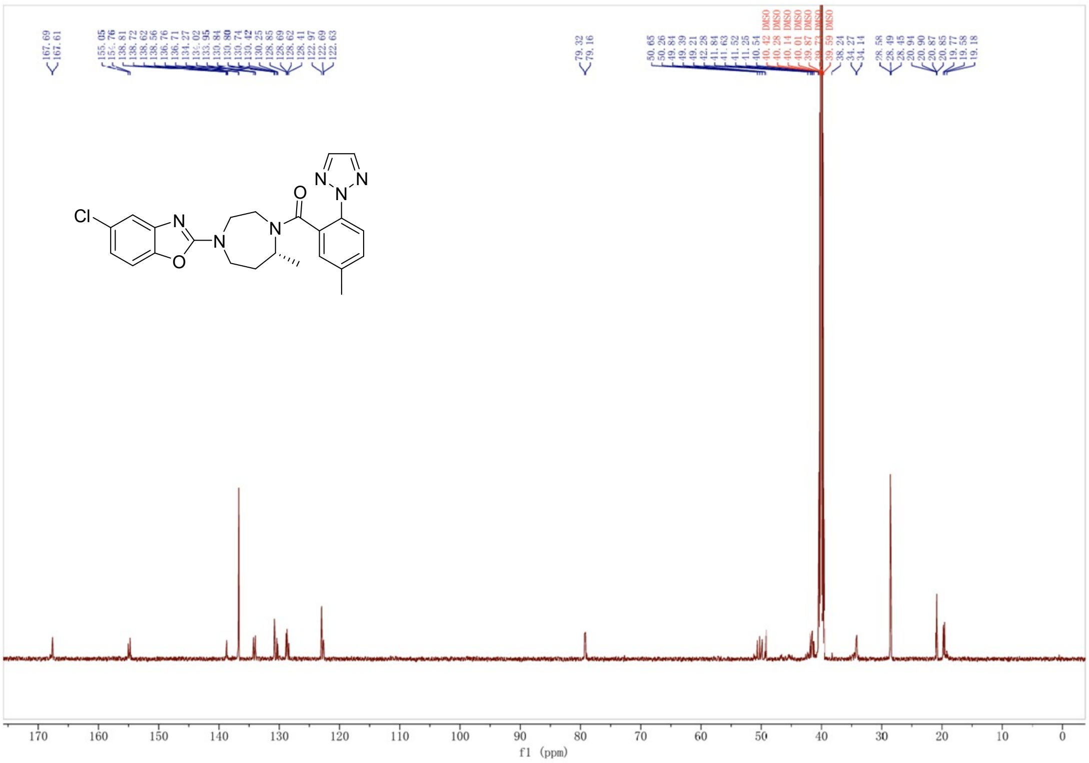

Supplementary materials for mass spectra of the compounds:

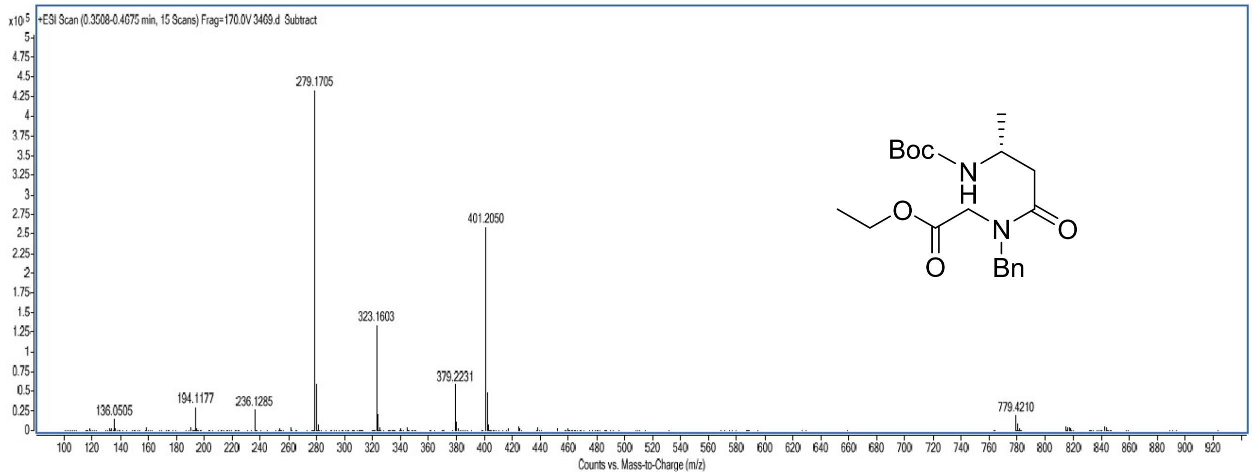

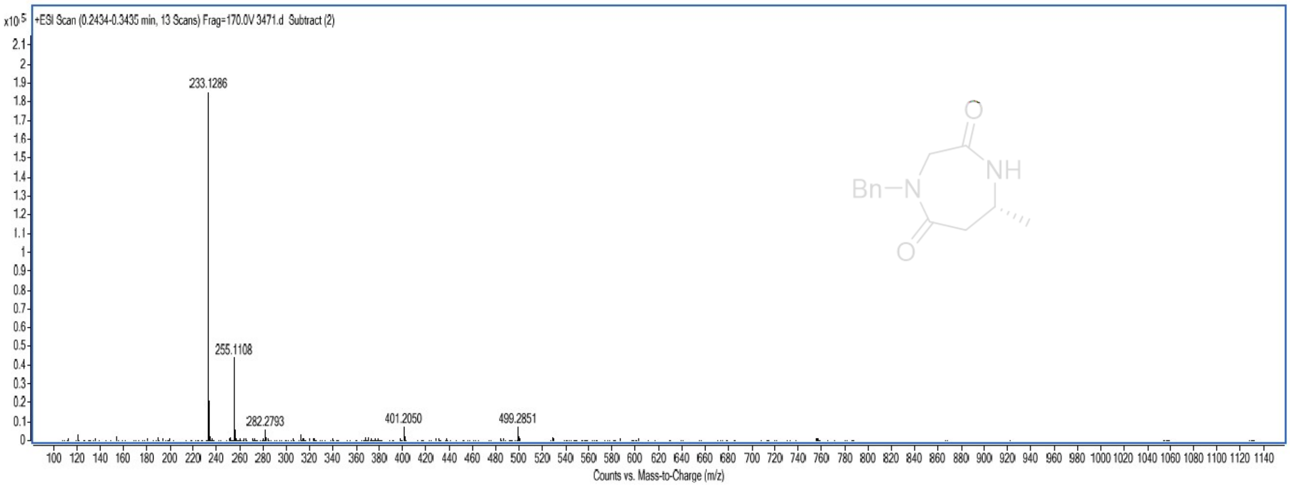

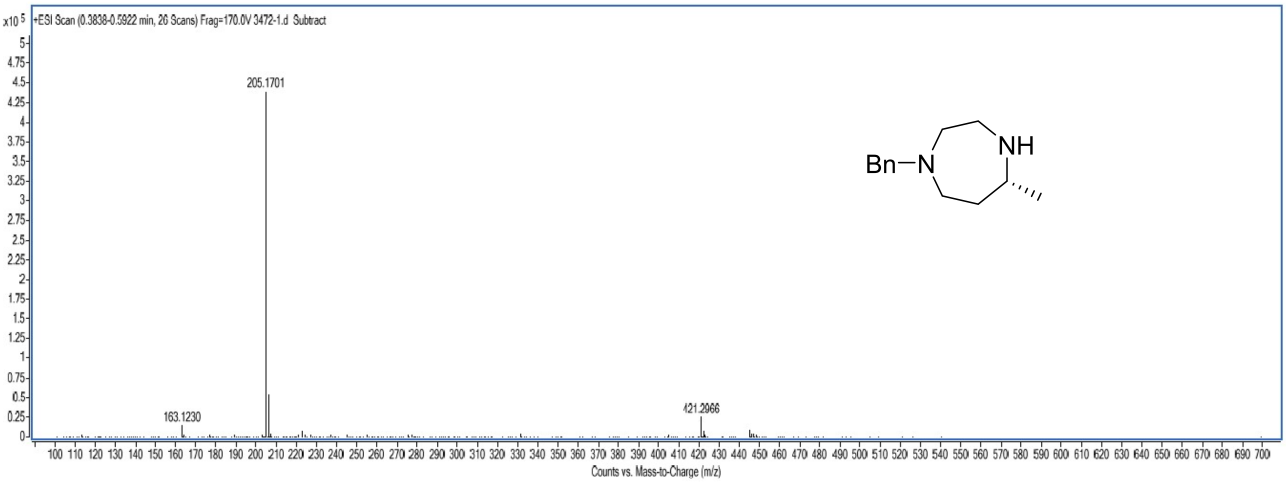

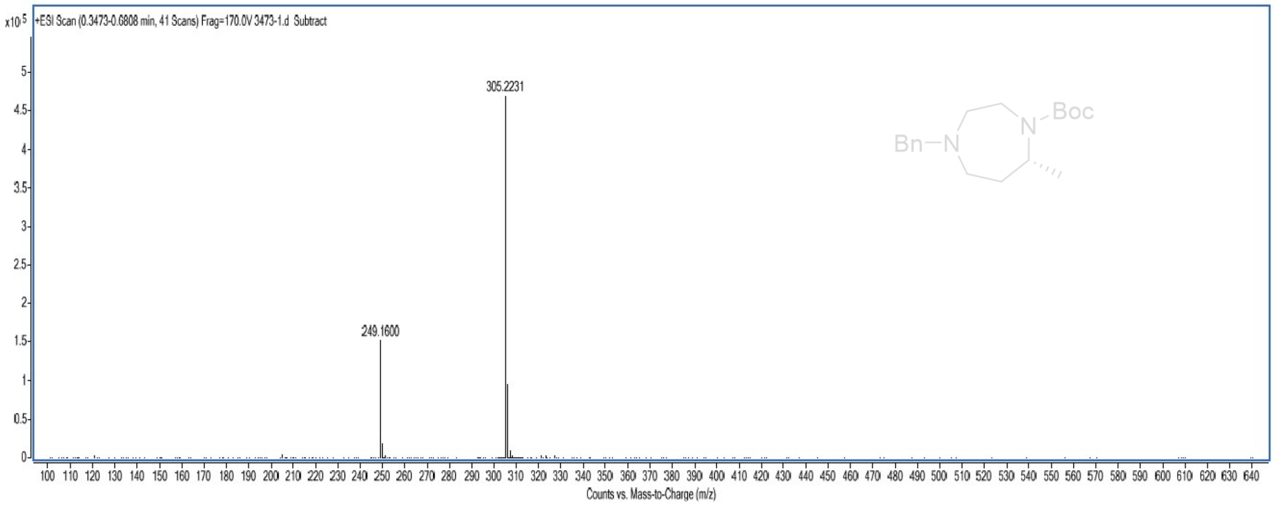

x10^5
+ESI Scan (0.3473-0.6808 min, 41 Scans) Frag=170.0V 3473-1.d Subtract
305.2231
249.1600
Counts vs. Mass-to-Charge (m/z)
Bn
N
N
Boc

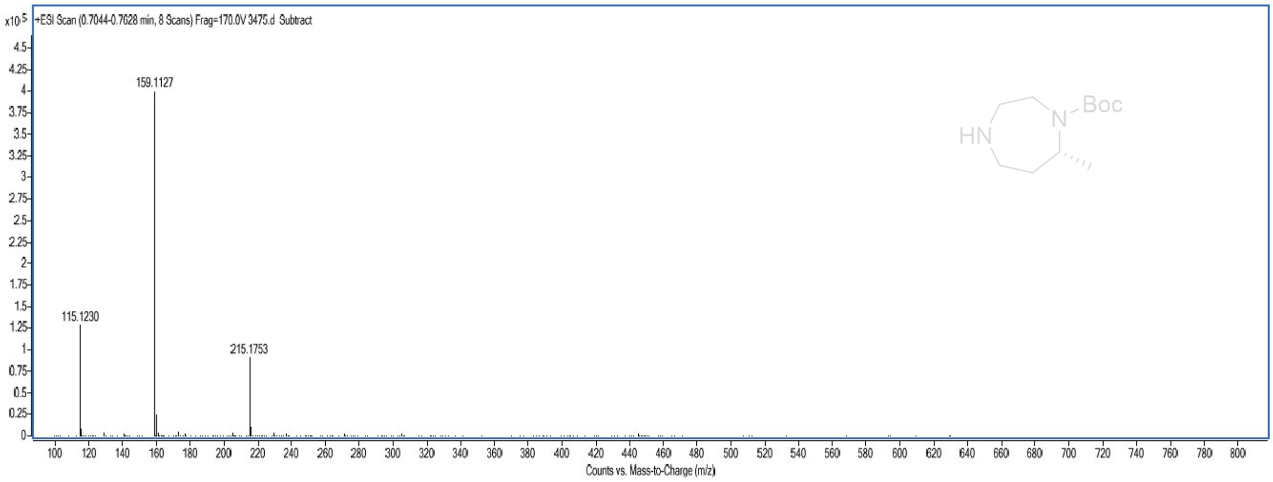

x10^5
+ESI Scan (0.7044-0.7628 min, 8 Scans) Frag=170.0V 3475.d Subtract
159.1127
115.1230
215.1753
Counts vs. Mass-to-Charge (m/z)
HN
N
Boc

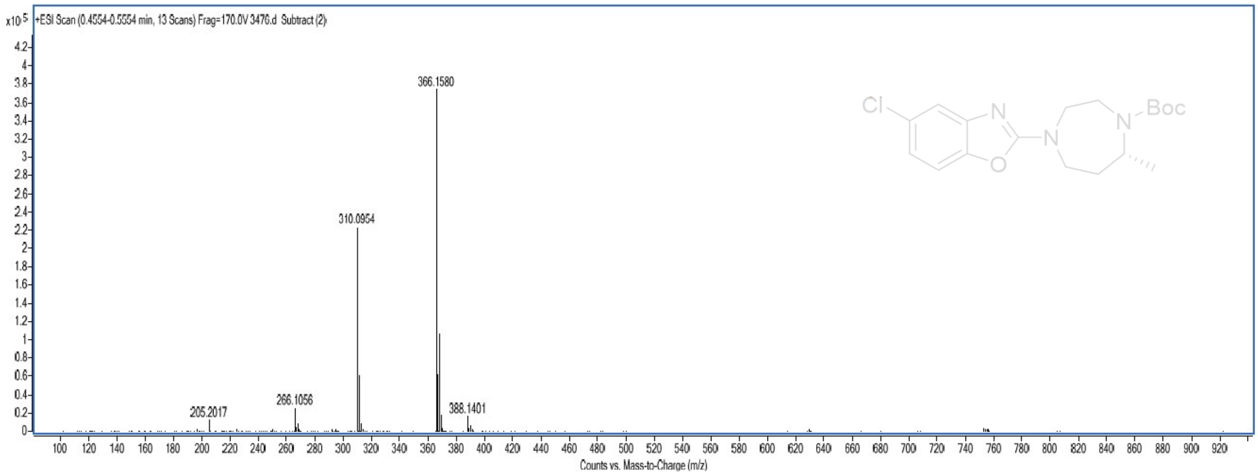

x10^5
+ESI Scan (0.4554-0.5554 min, 13 Scans) Frag=170.0V 3476.d Subtract (2)
366.1580
310.0954
266.1056
205.2017
388.1401
Counts vs. Mass-to-Charge (m/z)
Cl
N
O
N
N
Boc

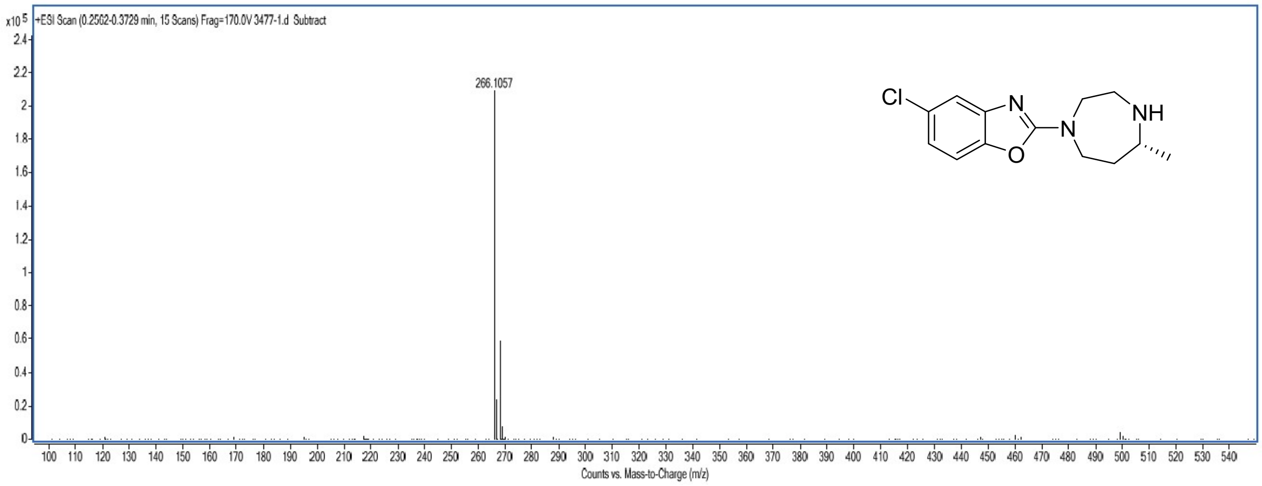

+ESI Scan (0.2502-0.3729 min, 15 Scans) Frag=170.0V 3477-1.d Subtract
266.1057
Cl
N
O
N
NH
Counts vs. Mass-to-Charge (m/z)

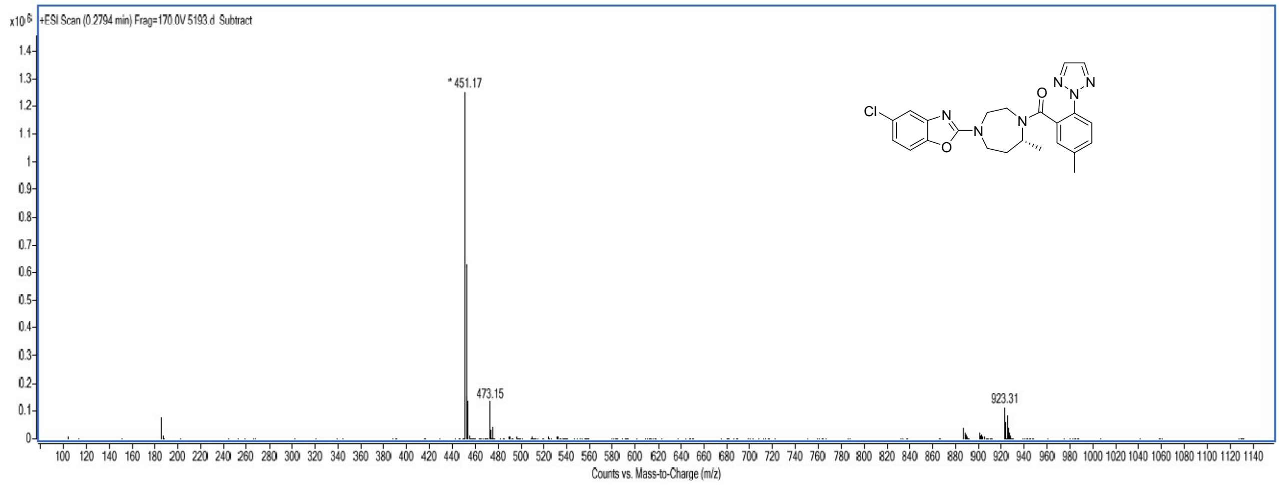

+ESI Scan (0.2794 min) Frag=170.0V 5193.d Subtract
* 451.17
473.15
923.31
Cl
N
O
N
N
O
N
N
N
Counts vs. Mass-to-Charge (m/z)
