# Supplementary material for: Optimized synthesis of suvorexant and determination of eight residual solvents by headspace gas chromatography
Source: RSC Adv. 2025 Oct 30;15(49):41597–607. doi: 10.1039/d5ra06779k (PMC12573263; doi:10.1039/d5ra06779k)
Supplement: RA-015-D5RA06779K-s002 [file RA-015-D5RA06779K-s002.pdf]

Supplementary Information (SI) for RSC Advances.
This journal is © The Royal Society of Chemistry 2025

supplement data

Other Synthetic Route	Synthetic Method	Key Features	Overall Yield& Largest Scale	Synthetic Steps (vs This Work)	AE（%）
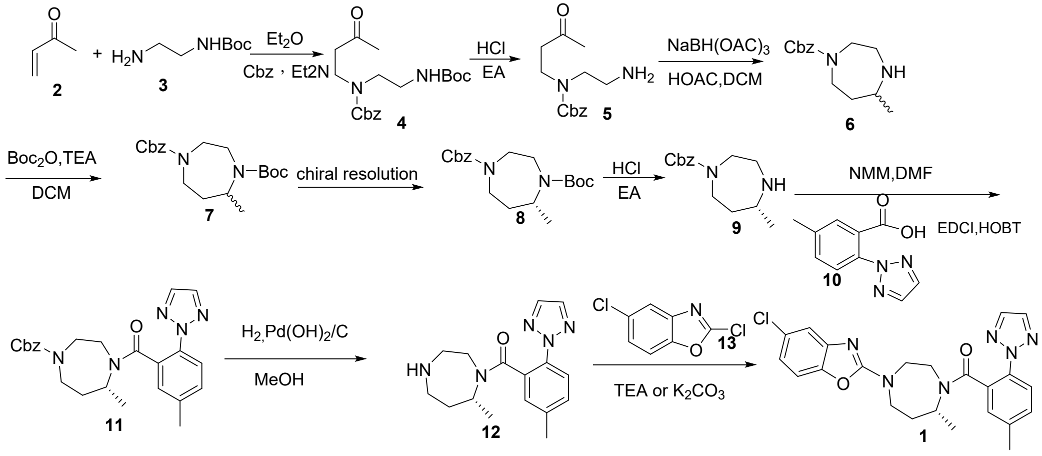	Chiral Column Separation and Resolution Method (Merck & Co.)	Chiral HPLC separation is required; it is suitable for laboratory use but not compatible with industrial scale-up.	11.00% & 200g	9/8	52.2

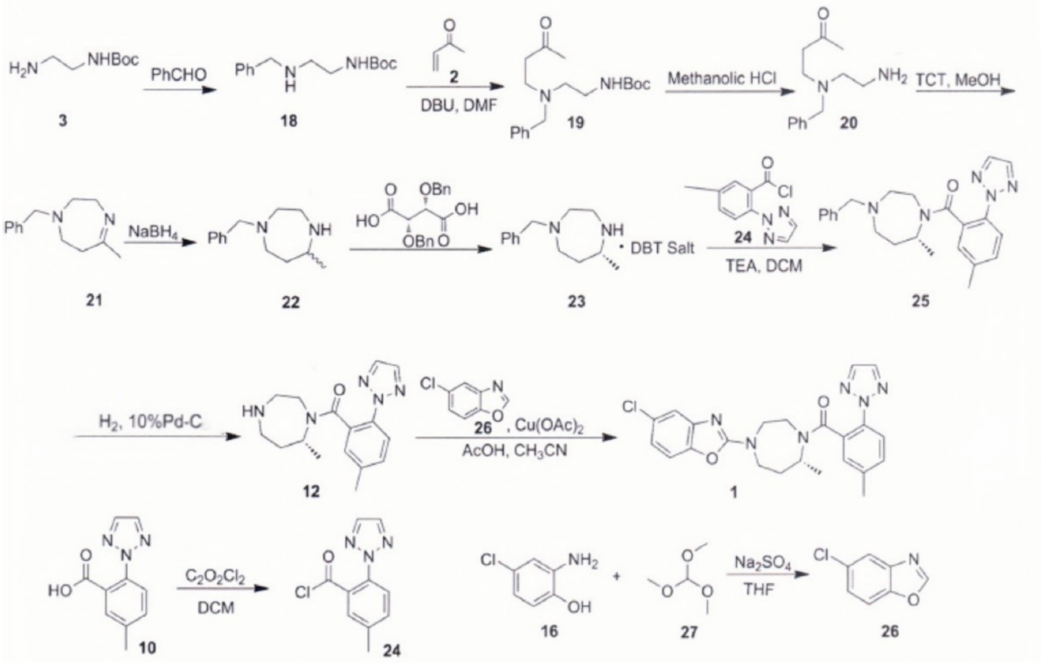	Chiral Column Separation and Resolution Method（Reddy's）	The protecting group was changed, but resolution is still required.	4.95% & 250g	9/8	60.3

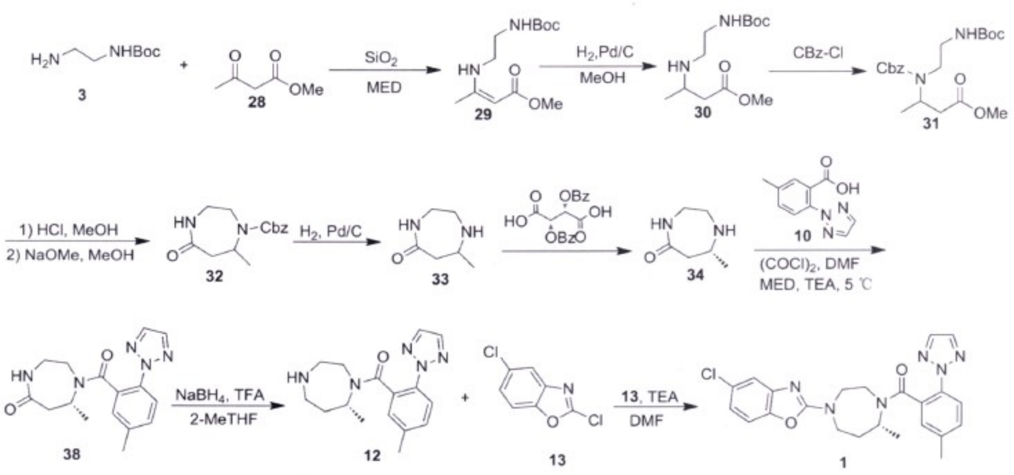	Chiral Column Separation and Resolution Method(Sandoz)	Protecting groups are used frequently, and the resolution yield is low.	<10.00% & 5g	9/8	45.2

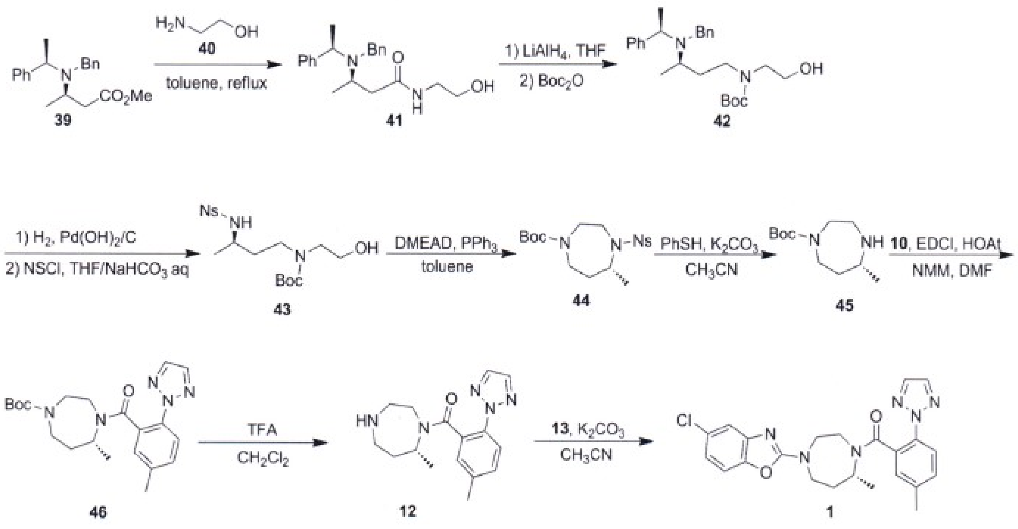	Chiral Starting Material Method (University of Toyama)	The number of protecting groups used is relatively large, and the raw materials need to be customized.	30.65% & 10g	8/8	50.7

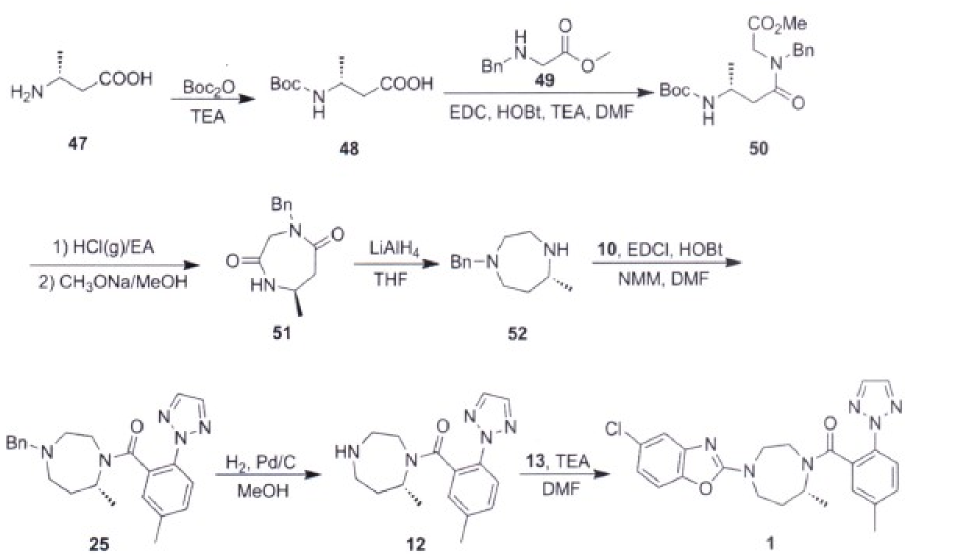	Chiral Starting Material Method （MINEHIRA D）	The enantiomeric excess (e.e.%) is greater than 99%, the yield of each step is high, and the process is industrially friendly.	56.00% & 15g	7/8	56.6
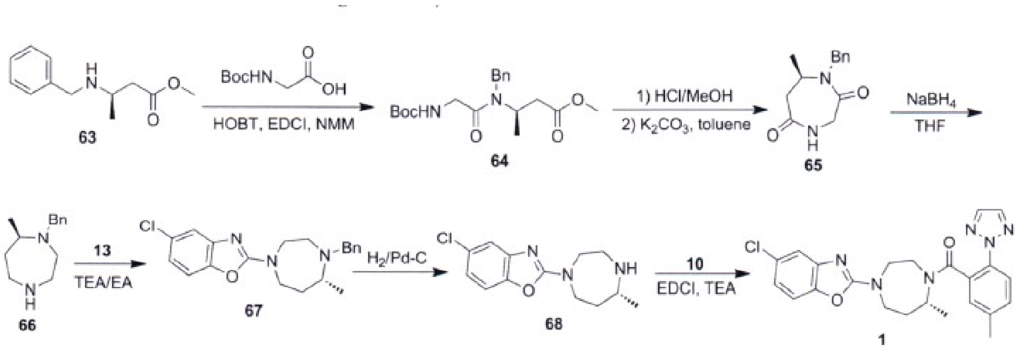	Chiral Starting Material Method （Shanghai Institute of Technology）	The yield is the highest, and the route is concise.	60.00% & 5g	6/8	58.2

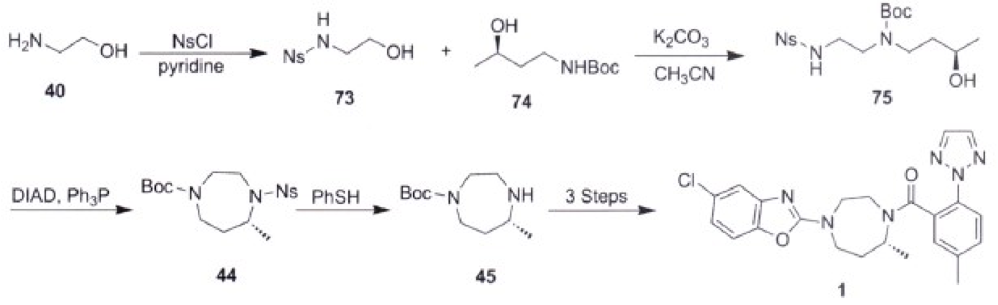	Chiral Starting Material Method （Ye Haiwei et al.）	The yield of the four-step reaction is 57.00%, and the overall yield is high.	40.50% & 15g	5/8	66.8
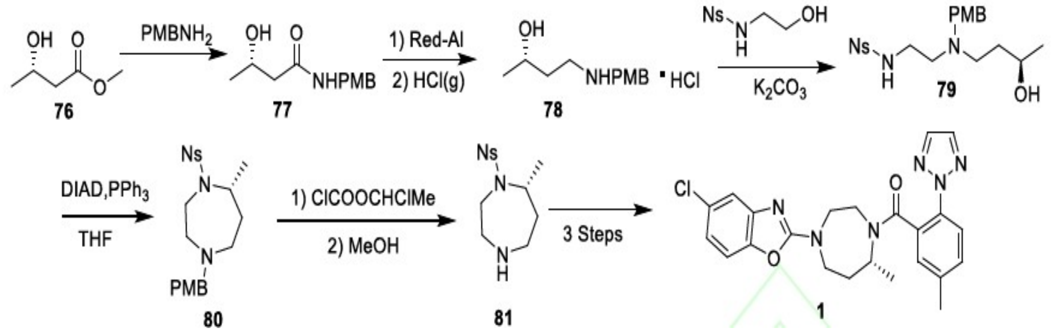	Chiral Starting Material Method (Translated by Qiu Kang et al.)	The post-treatment of the cyclization step is difficult.	7.50% & 5g	6/8	50.2

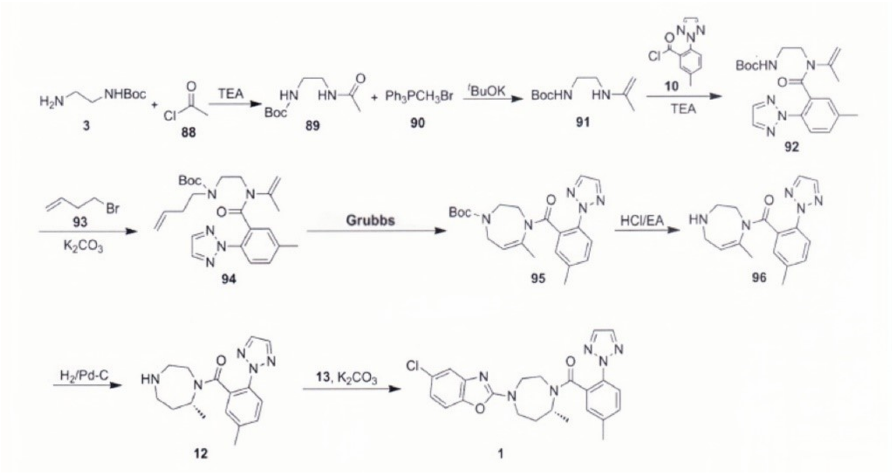	Grubbs Catalyst Method (Translated by Li Qiang et al.)	The use of Grubbs ruthenium catalysts comes with the drawback of high cost.	18.40% & 20g	9/8	51.3
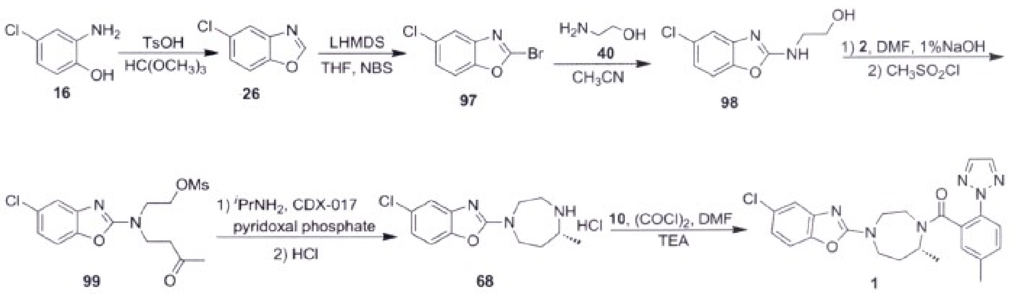	Biocatalytic Enzyme Method (Merck)	It avoids the use of halogenated solvents and heavy metals, but the enzyme catalyst is expensive.	43.00% & 25g	6/8	61.2

This Process Route	Synthetic Method	Key Features	Overall Yied & Largest Scale	Synthetic Steps	AE
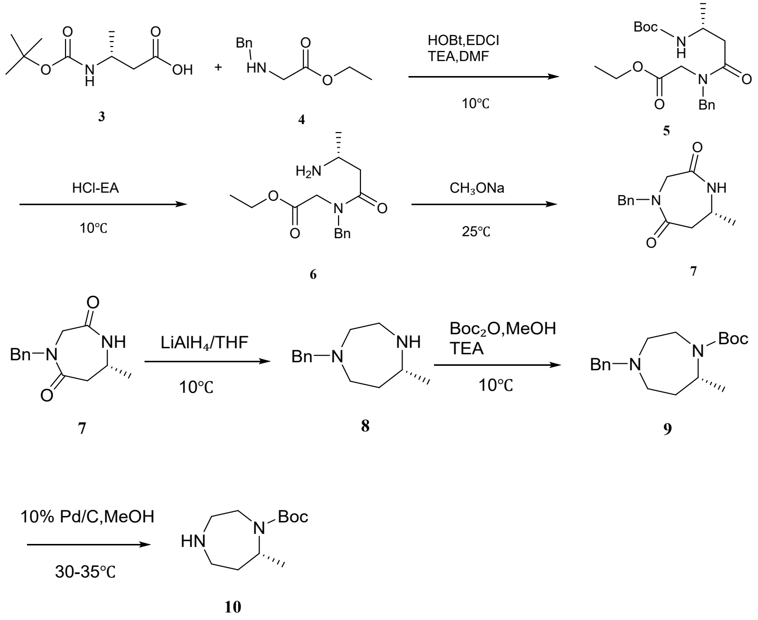	chiral starting material method （Chenshuo Jia et al.）	Employ the chiral starting material method to avoid chiral resolution	65%	8	52.3

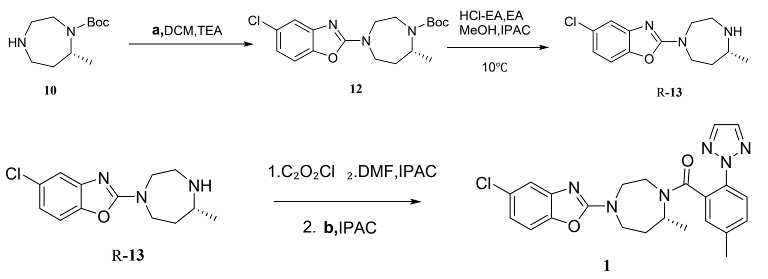
Boc
HN
a,DCM,TEA
10
Cl
N
O
Boc
12
HCl-EA,EA
MeOH,IPAC
10°C
NH
R-13
1.C2O2Cl 2.DMF,IPAC
2. b,IPAC
N
O
1
